# Supplementary material for: Spotiphy enables single-cell spatial whole transcriptomics across an entire section
Source: Nat Methods. 2025 Mar 12;22(4):724–36. doi: 10.1038/s41592-025-02622-5 (PMC11978521; doi:10.1038/s41592-025-02622-5)
Supplement: Supplementary file 1 — Supplementary Figs. 1–20, Methods and Tables 1–19. [file 41592_2025_2622_MOESM1_ESM.pdf]

nature methods

Supplementary information https://doi.org/10.1038/s41592-025-02622-5

Spotiphy enables single-cell spatial whole transcriptomics across an entire section

In the format provided by the authors and unedited

Content list

Supplementary Figures 1-20

Supplementary Methods

Supplementary Tables 1-19

Supplementary Figures

Supplementary Figure 1

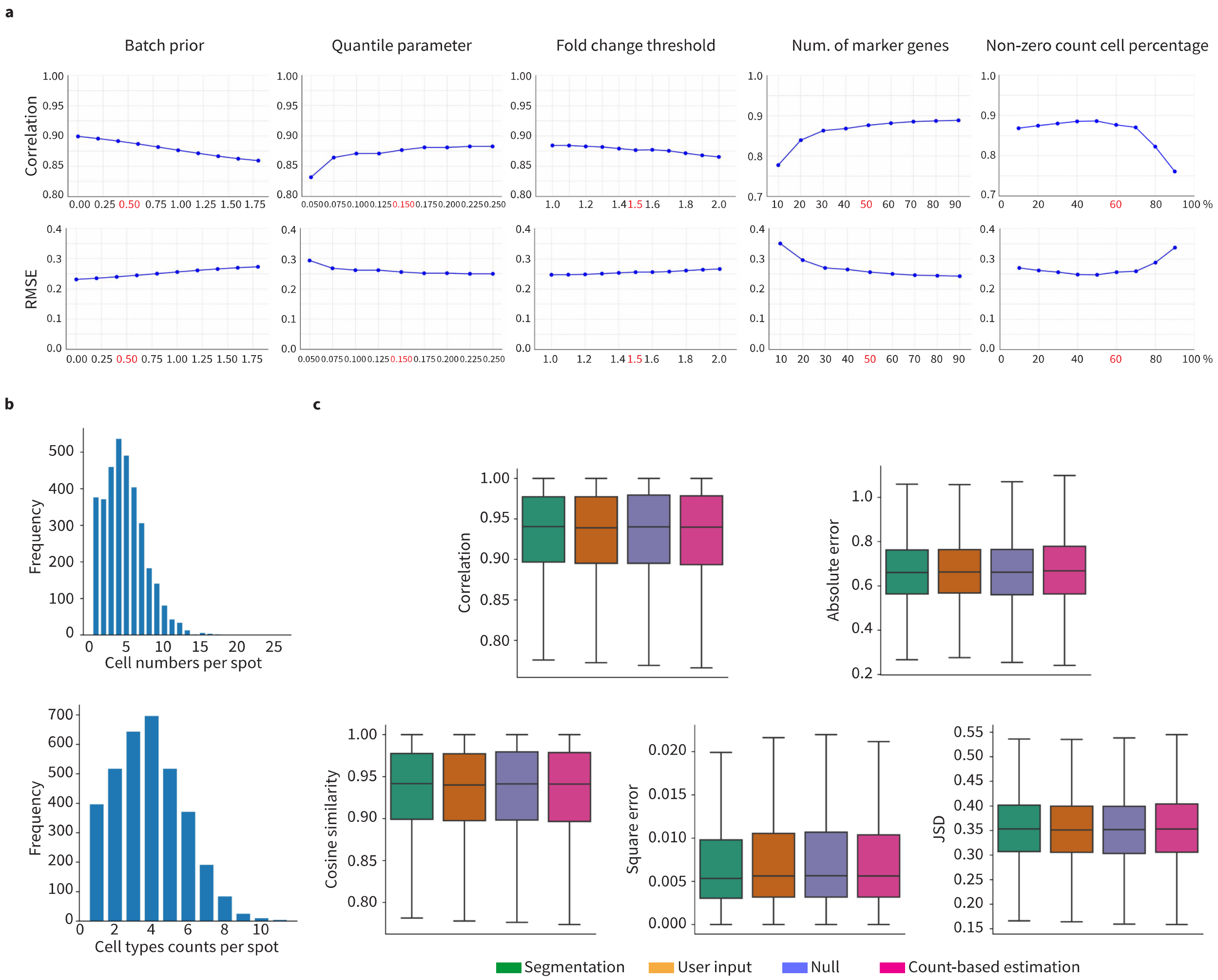

Supplementary Figure 1: Sensitivity analysis for hyperparameters. a, Sensitivity check of the five hyperparameters: Batch prior, quantile parameter, fold change threshold, number of marker genes, and non-zero count cell percentage. Correlation and RMSE are used as evaluation metrics. Default setting is marked as red. **b,** Histograms showing cell numbers and cell type counts per spot. **c,** Four opinions of Cell numbers (N values) of each spot provided by Spotiphy, and their impacts to decomposition outputs. Segmentation: N values come from StarDist's Segmentation results. User input: User manually input the N values. Null: User doesn't input any N values. Count-based estimation: N values come from total count-based estimation.

Boxplots illustrating the correlation, absolute error, cosine similarity, square error, and JSD for decomposition at each transcriptomic spot generated by each N input opinion. Boxplots are generated in the same manner as described in Figure 2. Each platform includes 3476 spots of AD sample.

Supplementary Figure 2

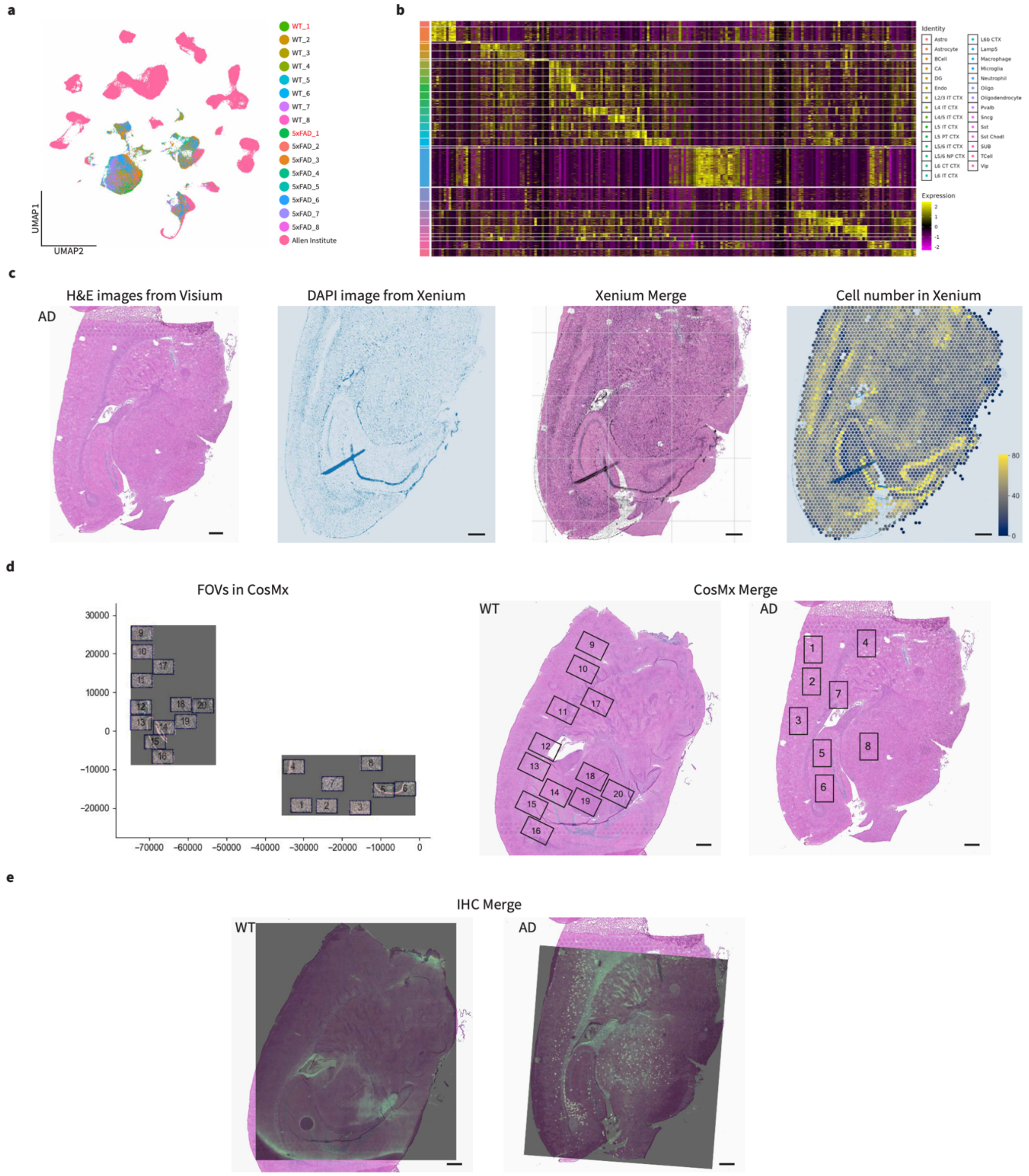

Supplementary Figure 2. scRNA reference construction and alignment of images from multiple ST datasets. a, UMAP projection of cells from scRNA-seq data used as mouse brain reference. Cells are labeled according to sample-of-origin. WT_1 and AD_1 were selected for further analysis in this study. **b,** Heatmap of expression of marker genes among 27 cell types. **c,** Alignment of Visium H&E image and

Xenium DAPI image of AD sample. **d**, Alignment of Visium H&E images and in-total 20 FOVs of CosMx DAPI images of WT and AD samples. **e**, Alignment of Visium H&E images and IHC staining images of WT and AD samples. Visium data includes one biological replicate each for WT and AD samples. Xenium data includes one biological replicate each for WT and AD samples. CosMx data includes one biological replicate each for WT and AD samples. Scale bar in **c-e**: 500 μm.

Supplementary Figure 3

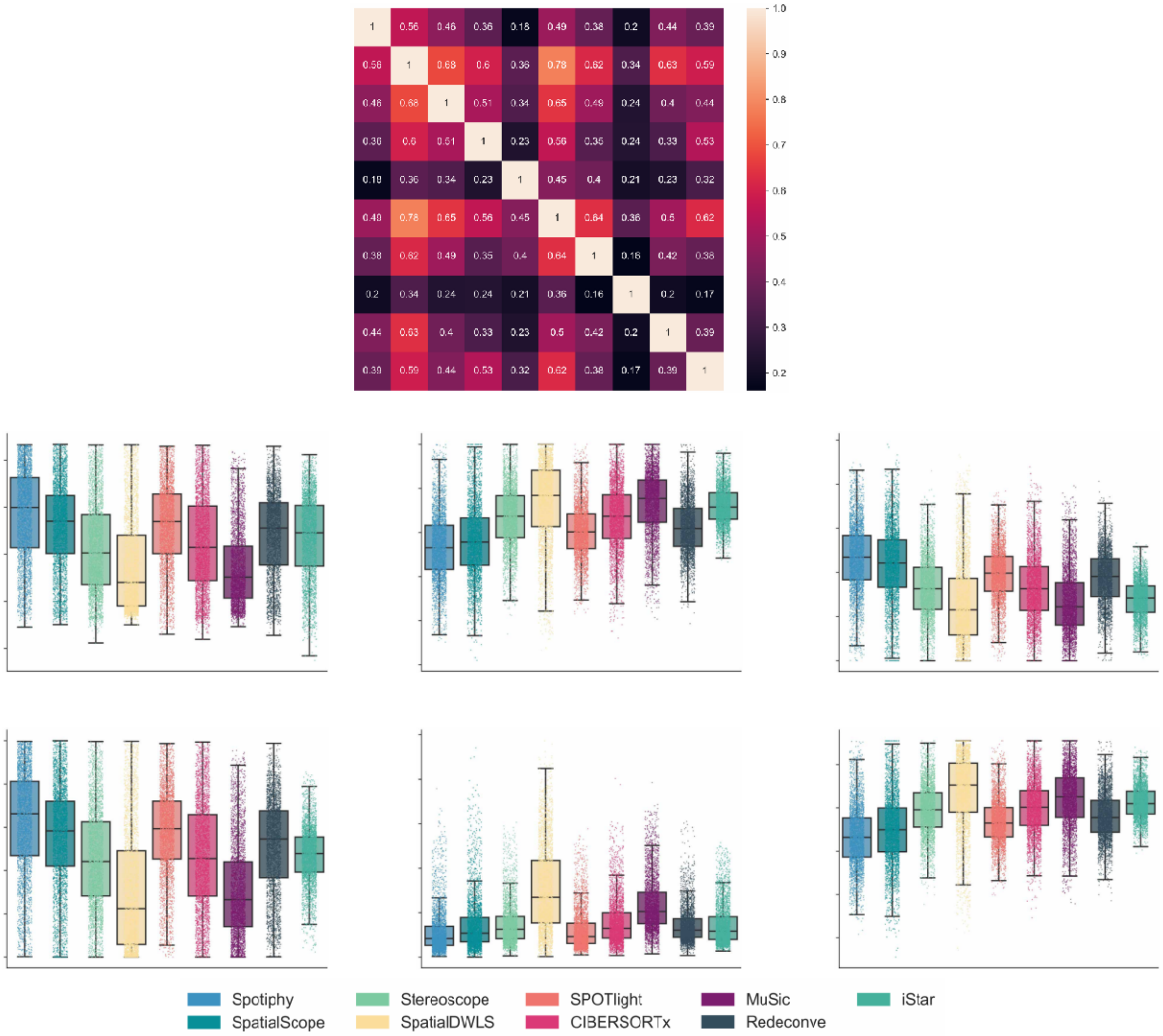

Supplementary Figure 3. Benchmarking Spotiphy's cellular deconvolution using matched Xenium data. a, Pearson correlation coefficient heatmap of cell-type proportions generated by Xenium, Spotiphy, and additional 8 methods selected for benchmarking. **b-g,** Box plots for correlation (**b**), absolute error (**c**), fraction of cells correctly mapped (**d**), cosine similarity (**e**), square error (**f**), and JSD (**g**) of the cell-type proportions for each transcriptomic spot generated by each method. Boxplots are generated in the same manner as described in Figure 2. Each platform includes 3476 spots of AD sample.

Supplementary Figure 4

a

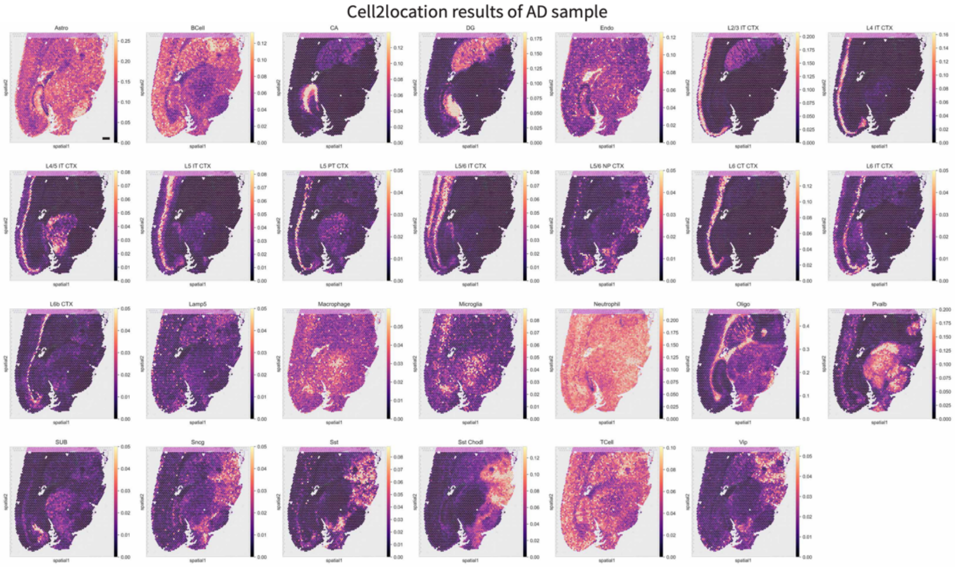

b

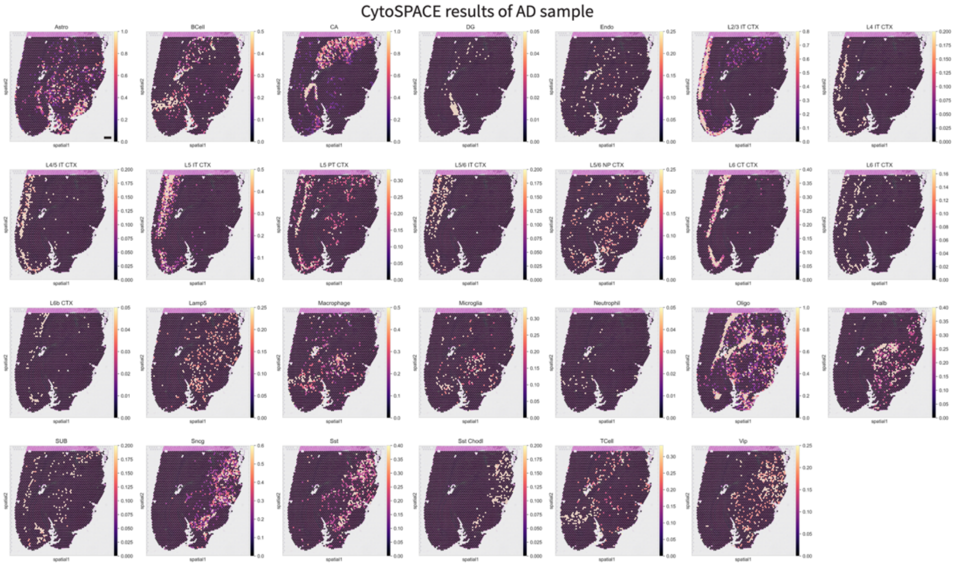

c

CARD results of AD sample

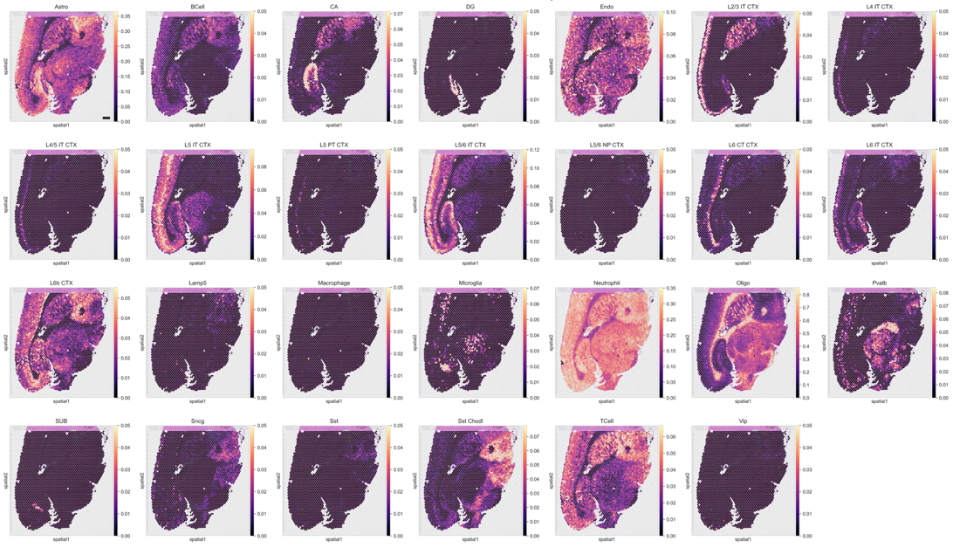

d

Tangram results of AD sample

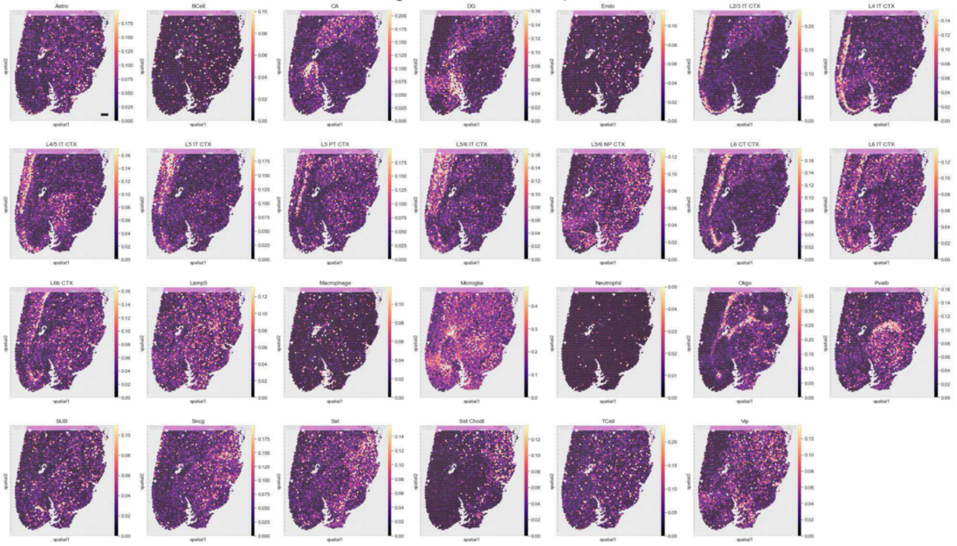

e

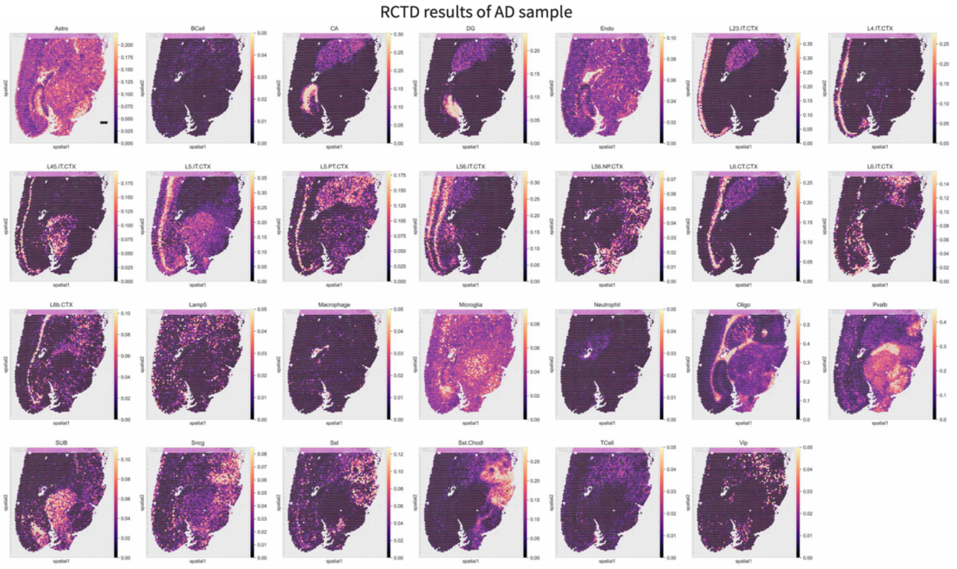

Supplementary Figure 4. Heatmaps depicting the proportion of 27 cell types generated by Cell2location (a), CytoSPACE (b), CARD (c), Tangram (d), and RCTD (e) across the histological section of AD mouse sample. Visium data of AD sample was used as test data. Scale bar: 500 μm.

Supplementary Figure 5

a

SpatialScope results of AD sample

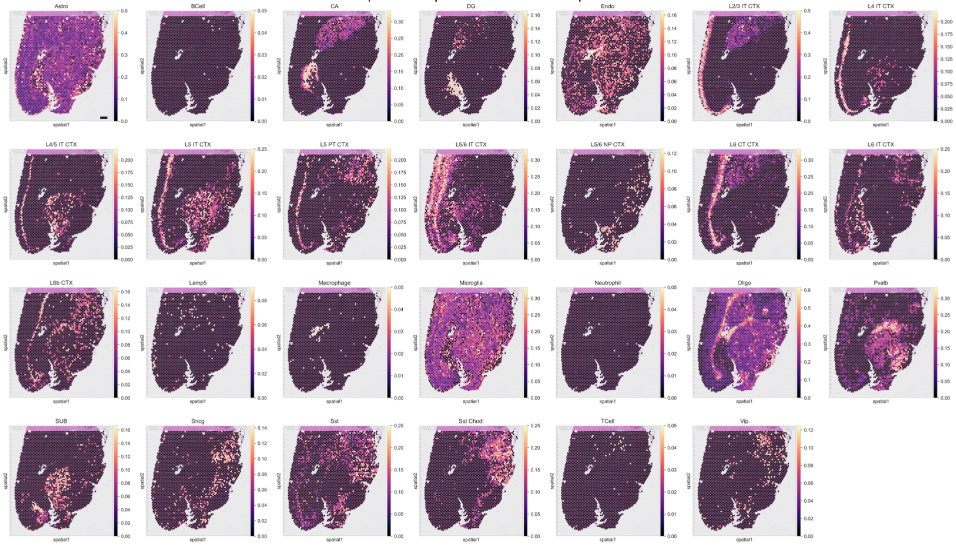

b

StereoScope results of AD sample

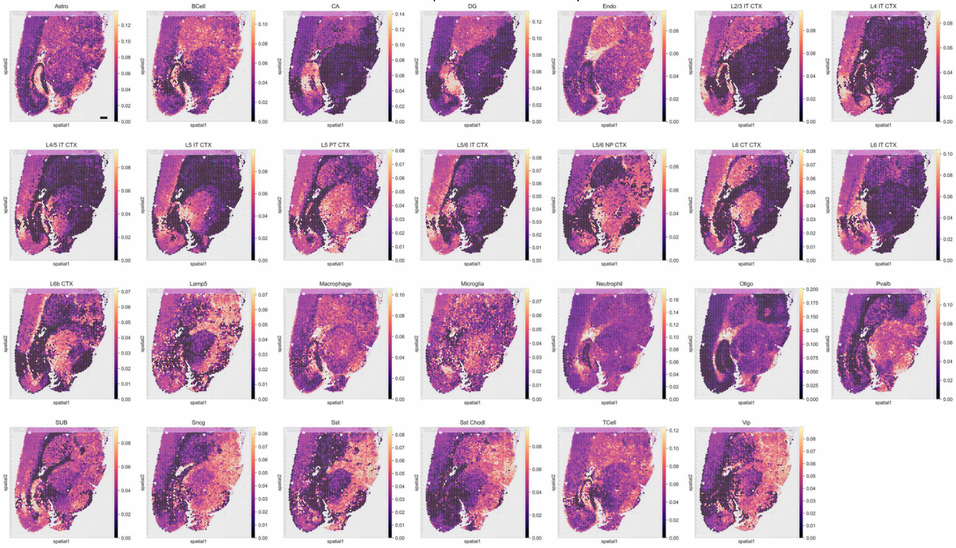

c

SpatialDWLS results of AD sample

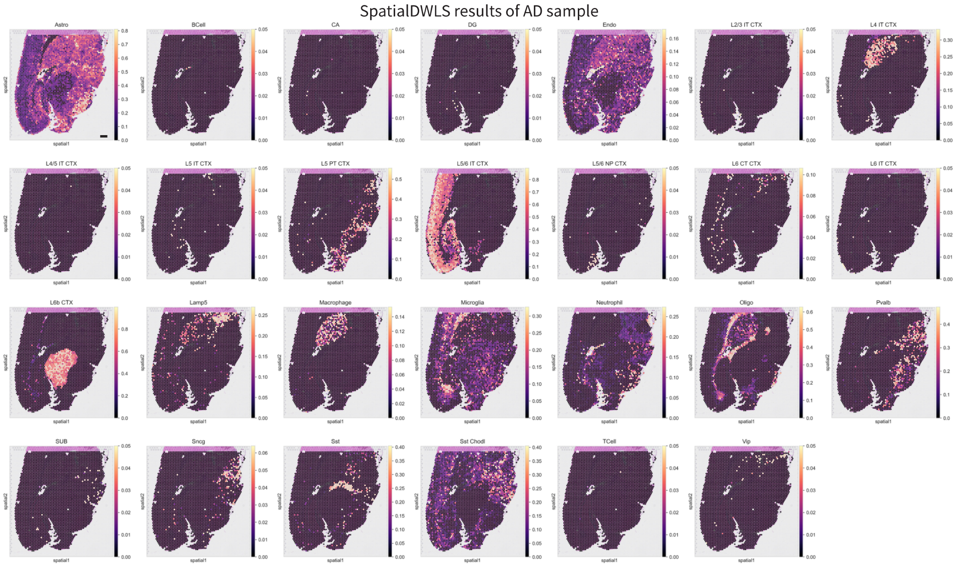

d

SPOTlight results of AD sample

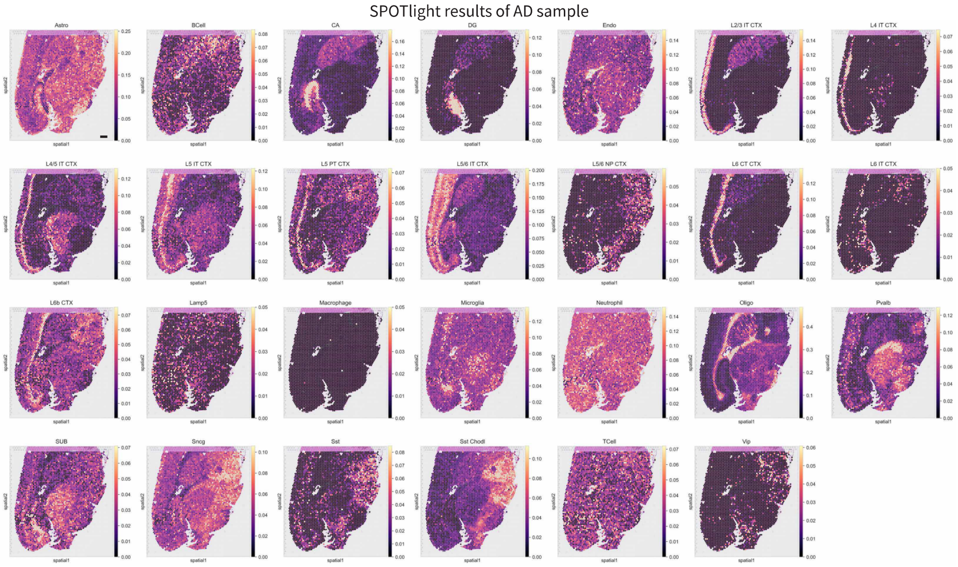

e

CIBERSORTx results of AD sample

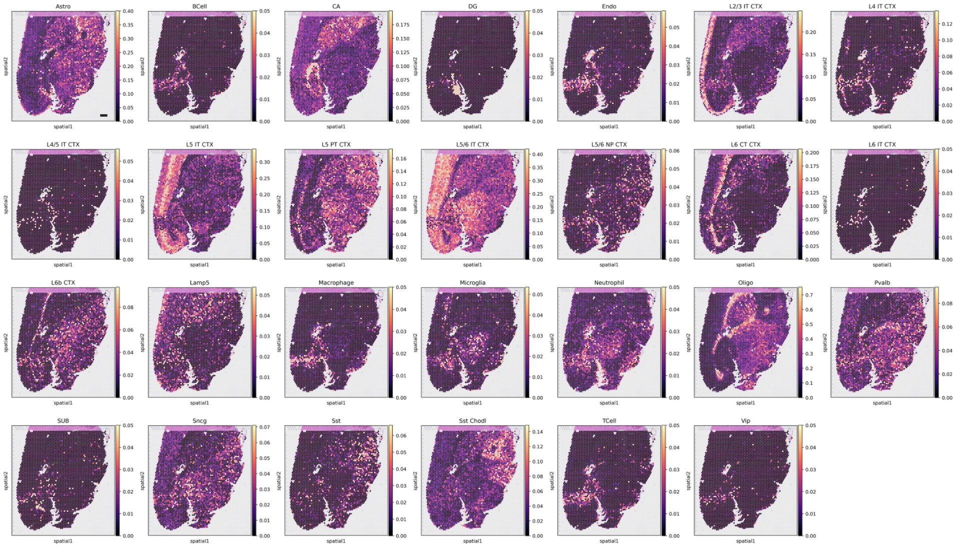

f

MuSiC results of AD sample

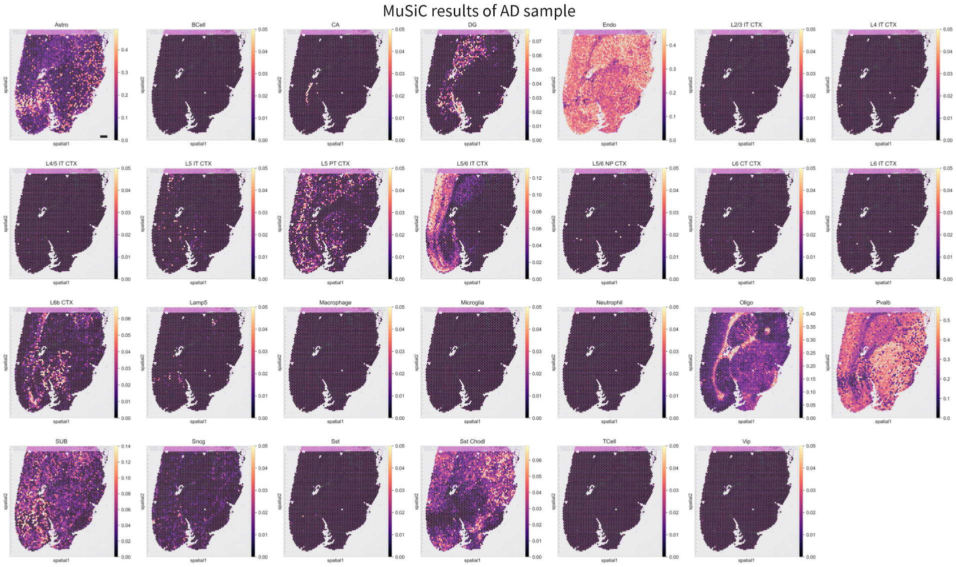

g

iStar results of AD sample

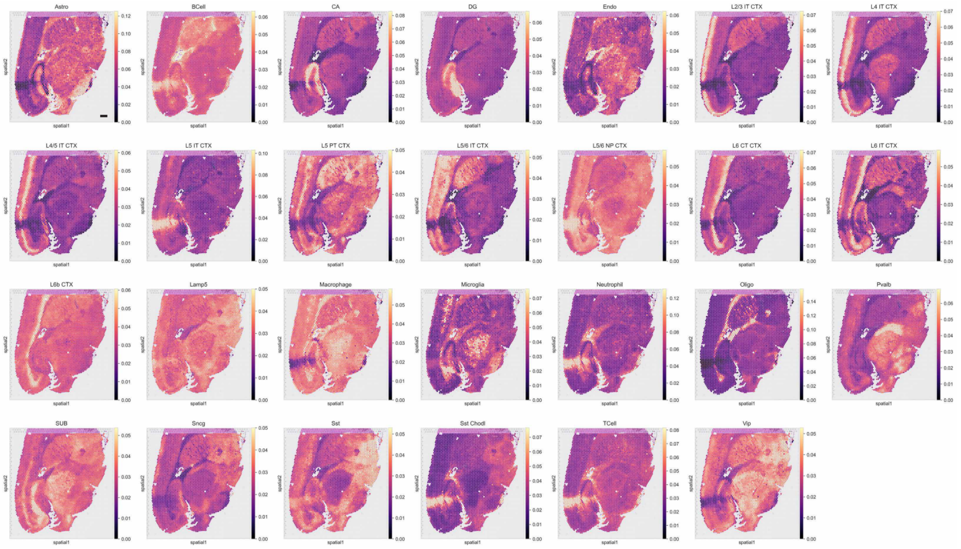

h

Redeconve results of AD sample

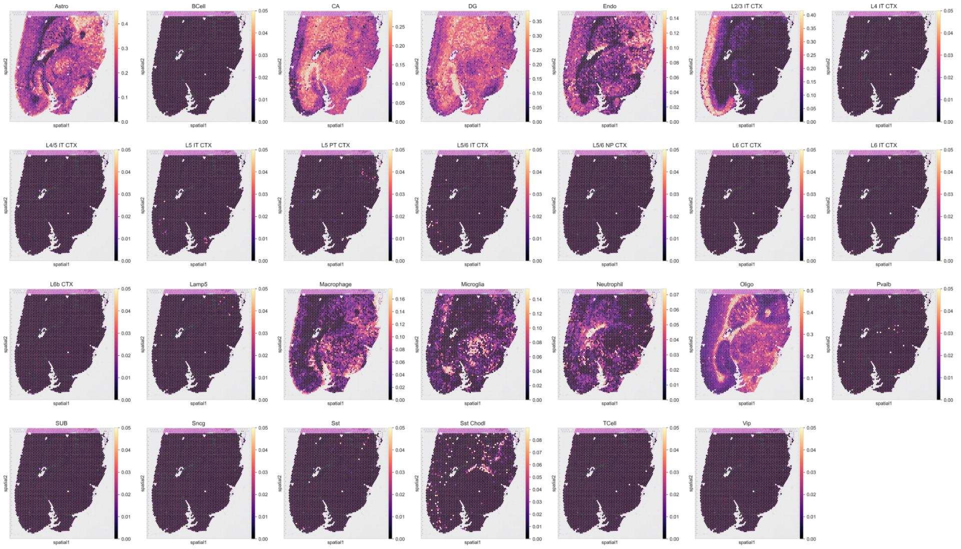

Supplementary Figure 5. Heatmaps depicting the proportion of 27 cell types generated by SpatialScope (a), Stereoscope (b), SpatialDWLS (c), SPOTlight (d), CIBERSORTx (e), MuSiC (f), iStar (g), and Redeconve (h) across the histological section of AD mouse sample. Visium data of AD sample was used as test data. Scale bar: 500 µm.

Supplementary Figure 6

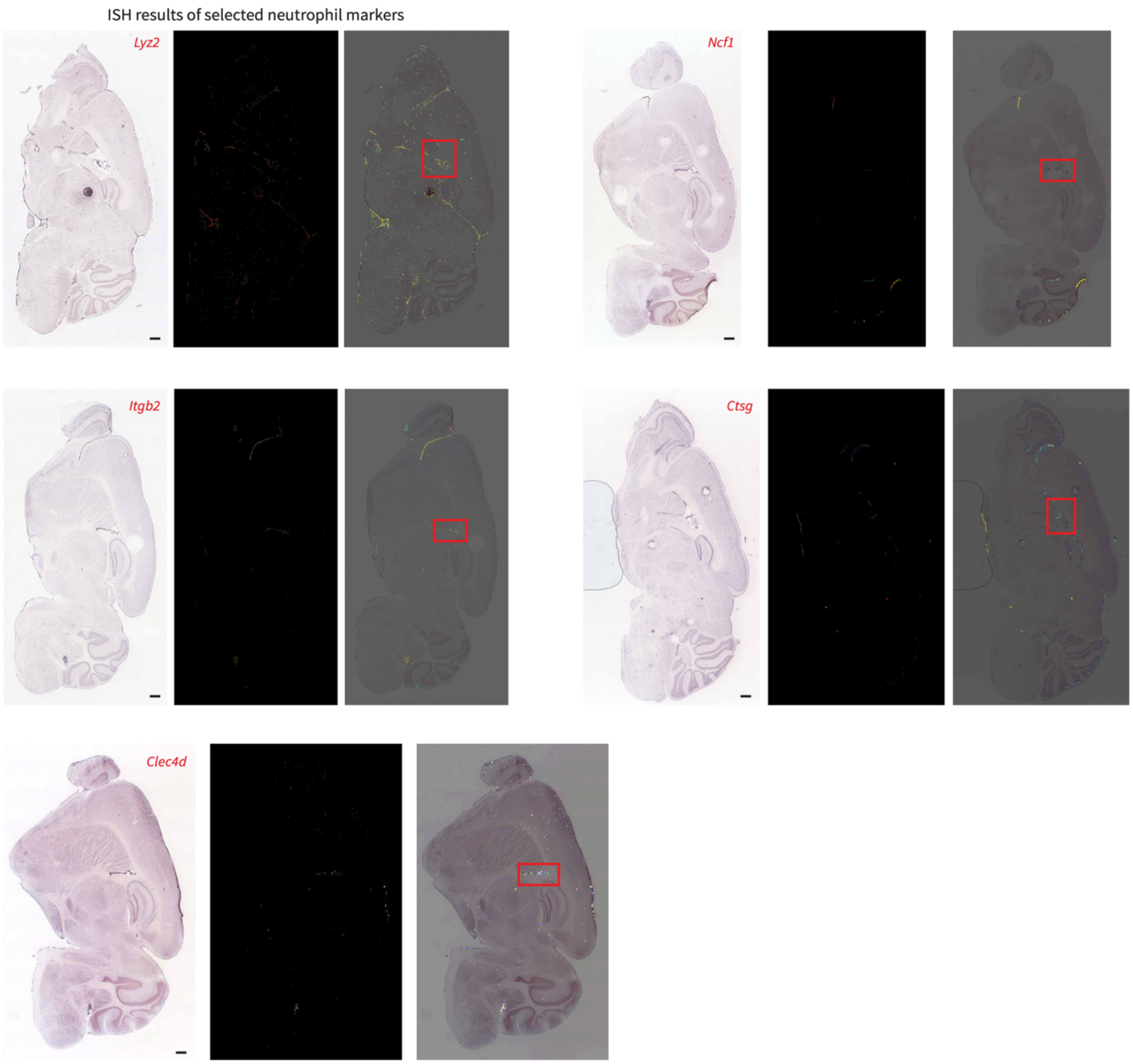

Supplementary Figure 6. In situ hybridization (ISH) images of selected neutrophil markers from Allen Institute Atlas. *Lyz2 (100055046), Ncf1 (70546251), Itgb2 (77464984), Ctsg (69608226),* and *Clec4d (71764721)* are commonly used markers for neutrophils. Left panel is ISH result, middle panel is expression result (fluorescence), right panel is the merged result. Red box showed the positive signals around the ventricle. Data were downloaded from Allen Mouse Brain Atlas (mouse.brain-map.org) with one replicate for each panel. Scale bar: 500 μm.

Supplementary Figure 7

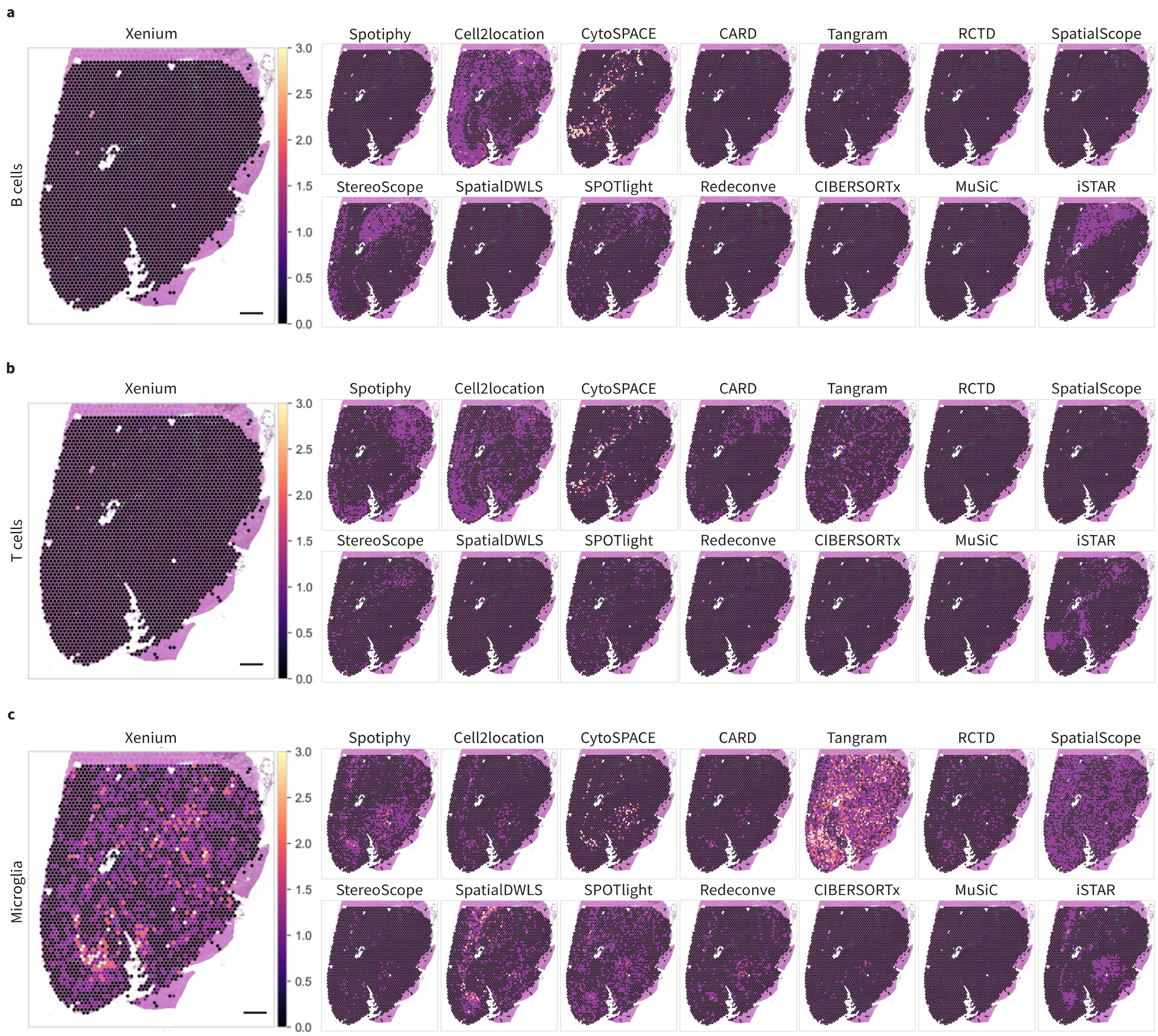

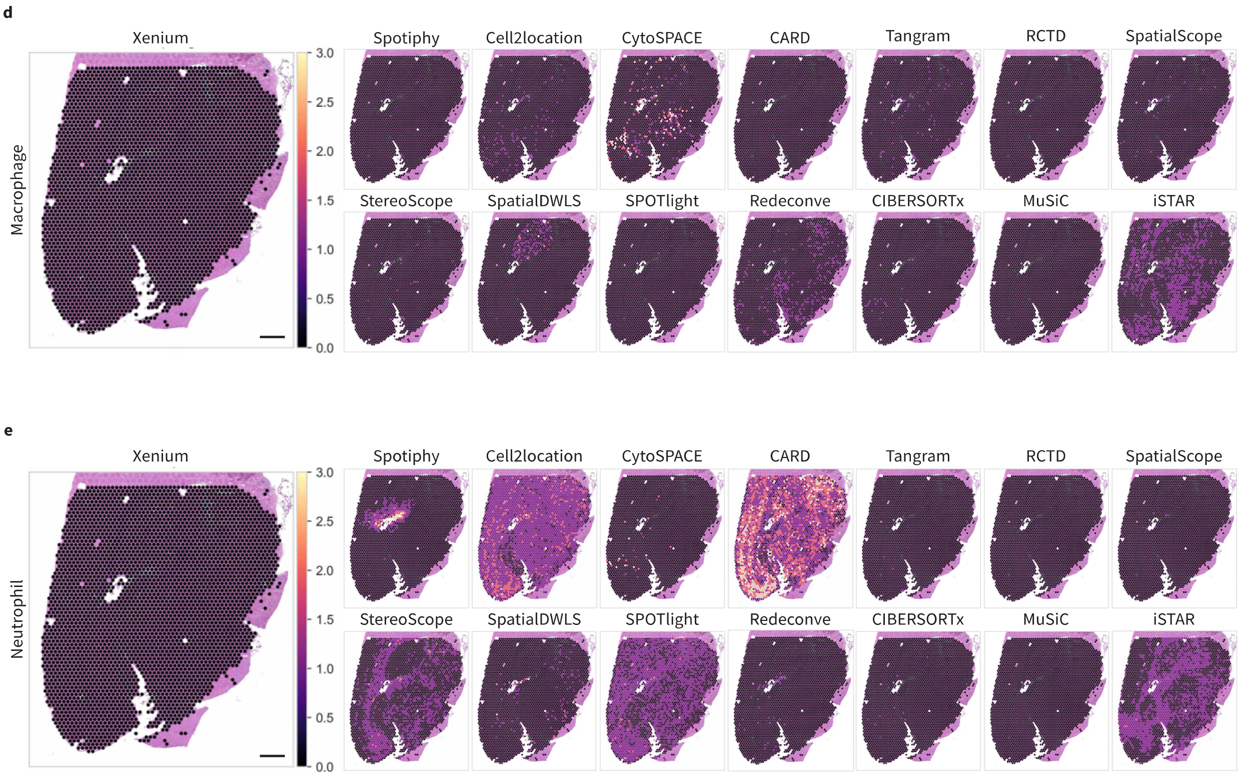

Supplementary Figure 7. Heatmaps depicting the absolute number of B cells (a), T cells (b), Microglia (c), Macrophage (d), and Neutrophils (e) generated by 14 deconvolution methods across the histological section of AD mouse sample. Scale bar: 500 μm.

Supplementary Figure 8

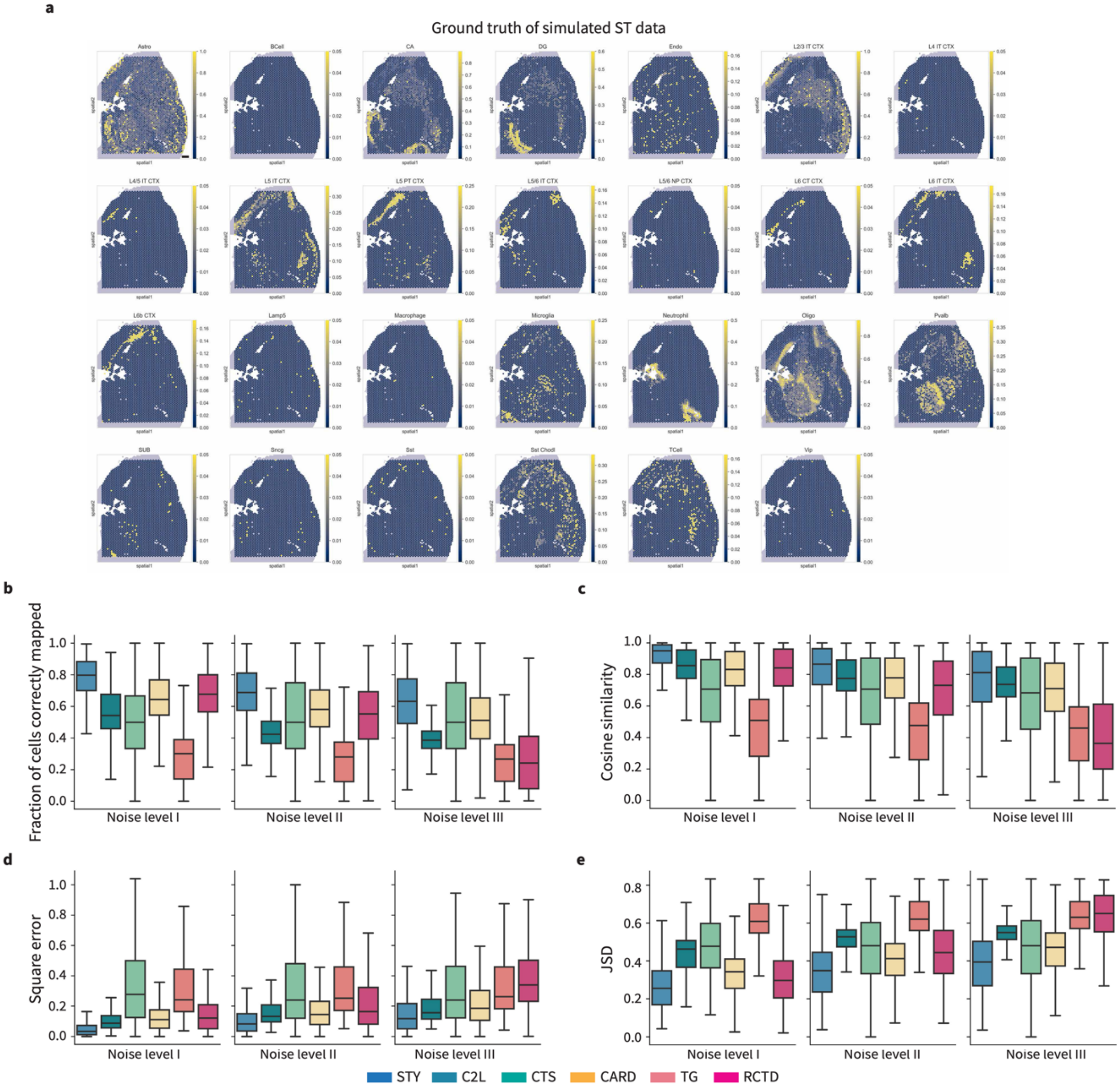

Supplementary Figure 8. The ground truth of simulated ST data and performance evaluation of methods including Spotiphy using simulated Visium data. a, Heatmaps depicting the ground truth of proportion of 27 cell types from simulated ST data across the histological section. Scale bar: 500 µm. **b-e,** Box plots for fraction of cells correctly mapped (**b**), cosine similarity (**c**), square error (**d**), and JSD (**e**) of the cell-type proportions for each transcriptomic spot generated by each method. Boxplots are generated in the same manner as described in Figure 2. Each platform includes 3476 spots of AD sample.

Supplementary Figure 9

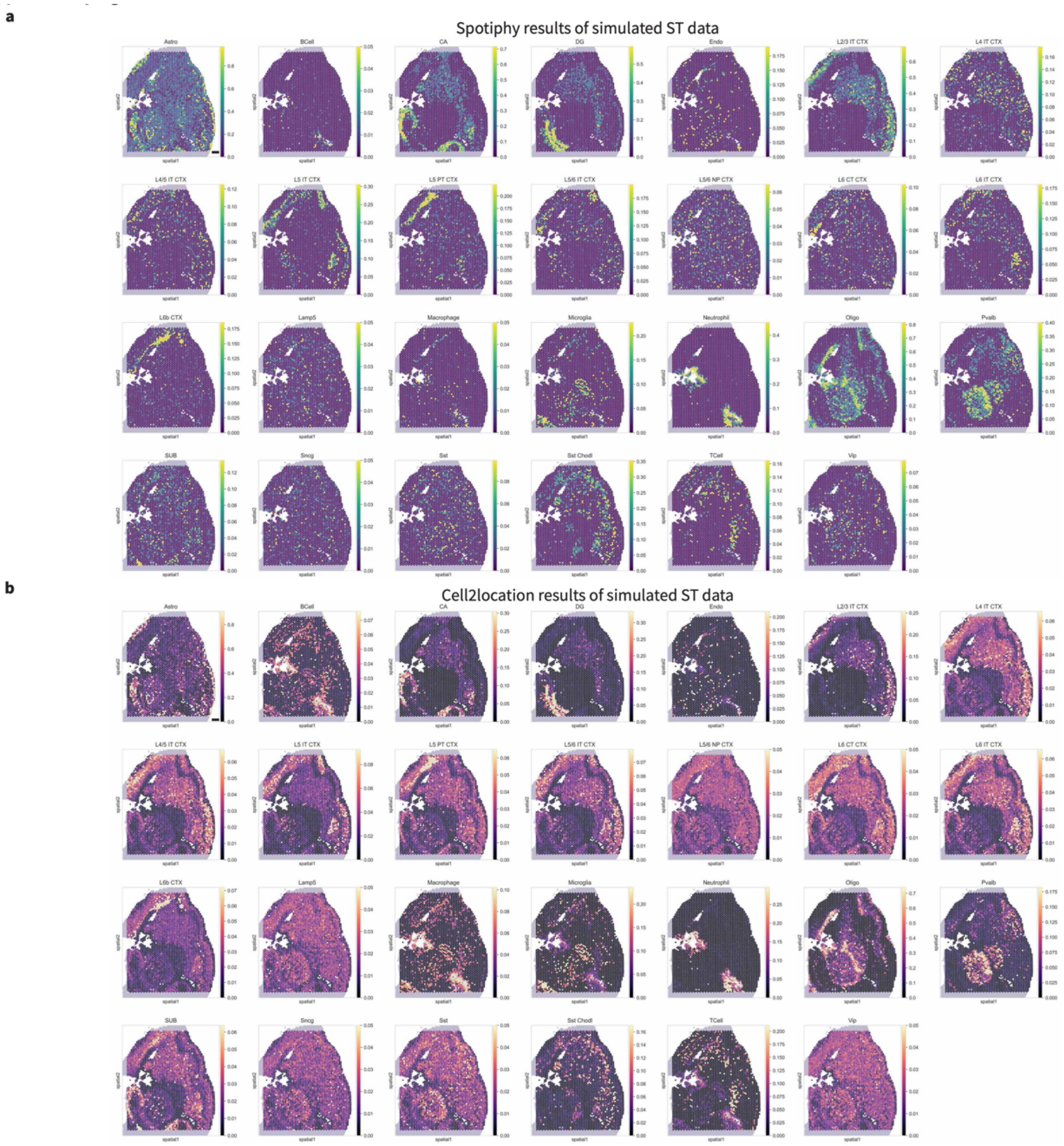

c

CytoSPACE results of simulated ST data

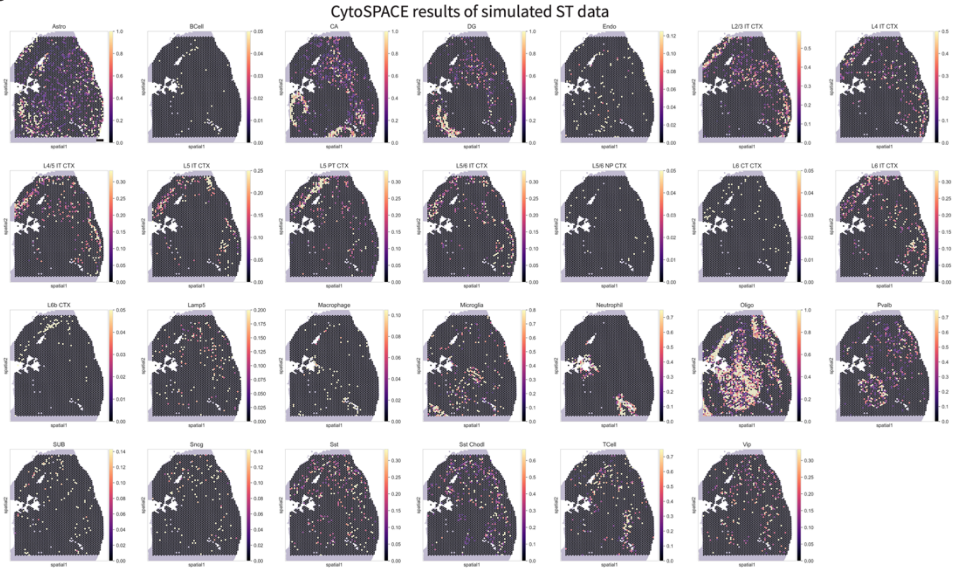

d

CARD results of simulated ST data

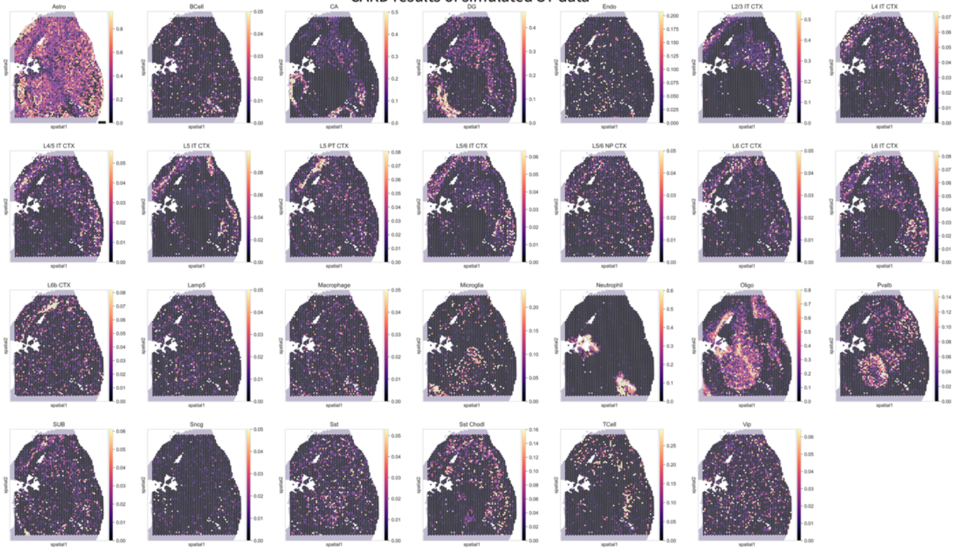

e

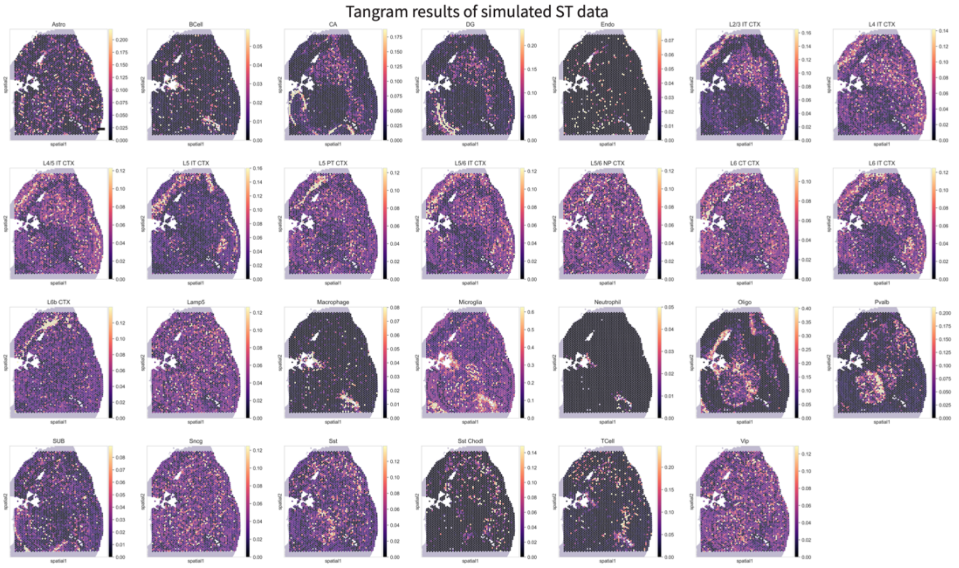

f

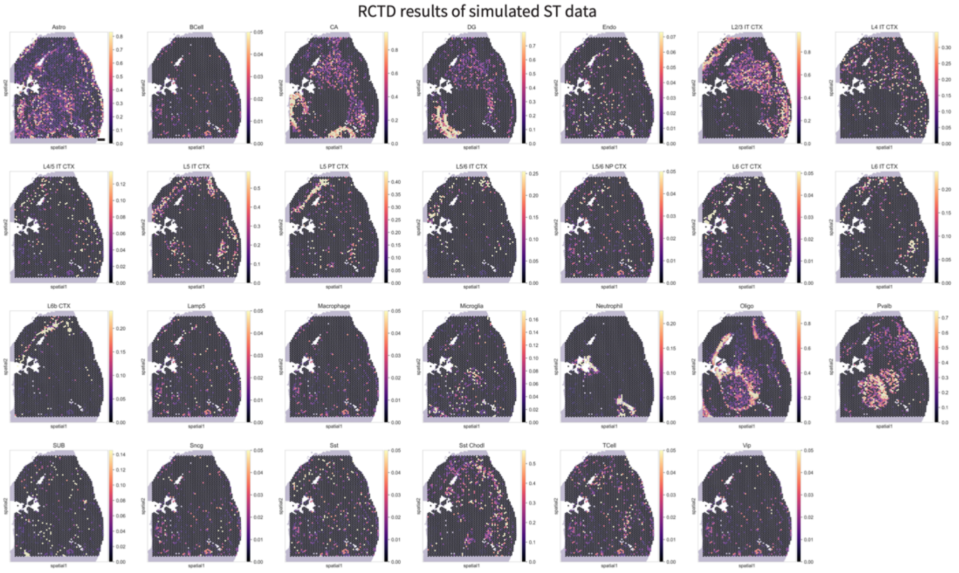

Supplementary Figure 9. Heatmaps depicting the proportion of 27 cell types generated by Spotiphy (a), Cell2location (b), CytoSPACE (c), CARD (d), Tangram (e), and RCTD (f) across the histological section of simulated Visium data. Scale bar: 500 μm.

Supplementary Figure 10

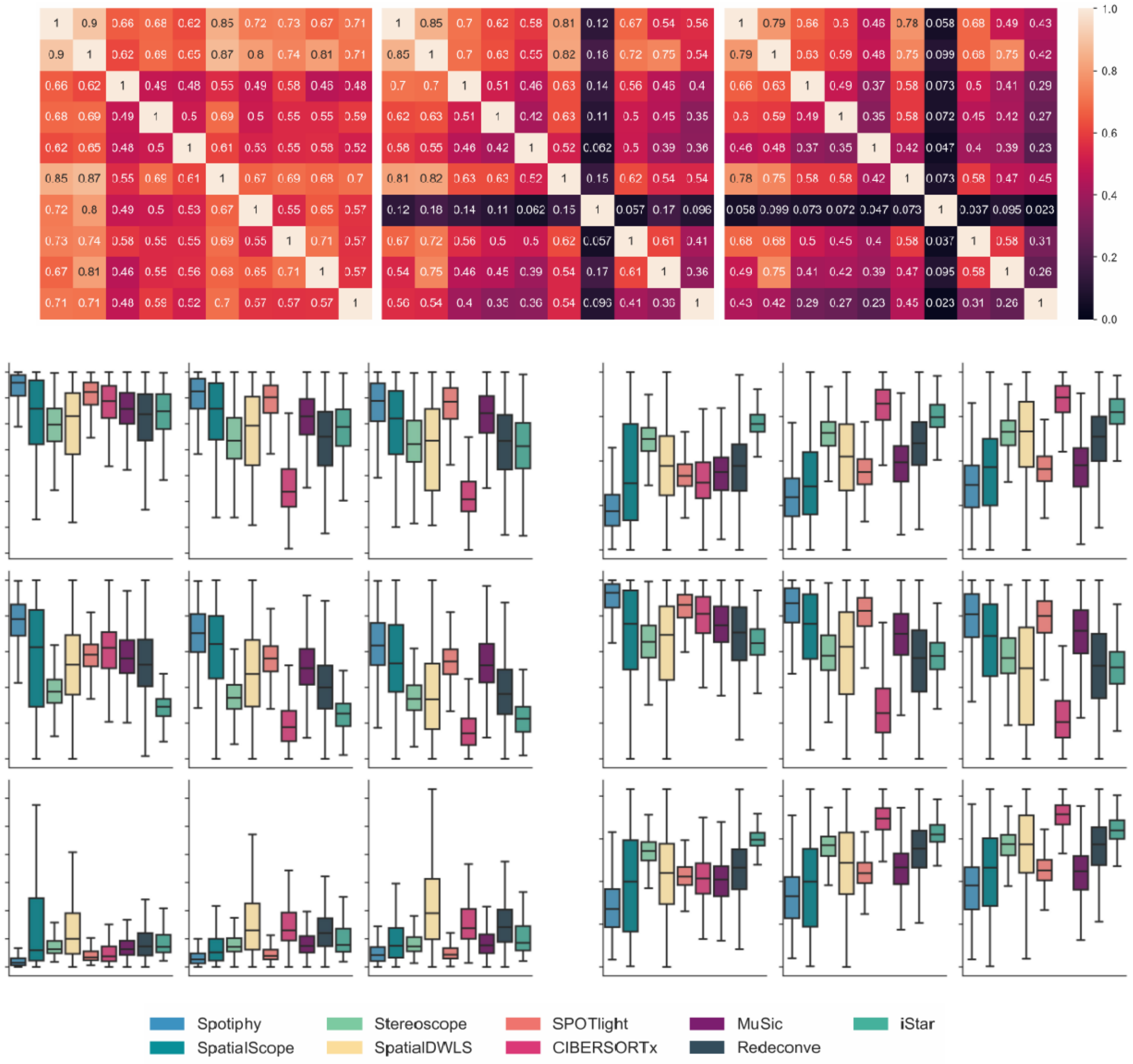

Supplementary Figure 10. The performance evaluation of additional methods using simulated Visium data. a, Pearson correlation coefficient heatmap of cell-type proportions generated by the simulated Visium (Ground truth), Spotiphy, and additional 8 methods selected for benchmarking. **b-g,** Box plots for correlation (**b**), absolute error (**c**), fraction of cells correctly mapped (**d**), cosine similarity (**e**), square error (**f**), and JSD (**g**) of the cell-type proportions for each transcriptomic spot generated by each method. Boxplots are generated in the same manner as described in Figure 2. Each platform includes 3476 spots of AD sample.

Supplementary Figure 11

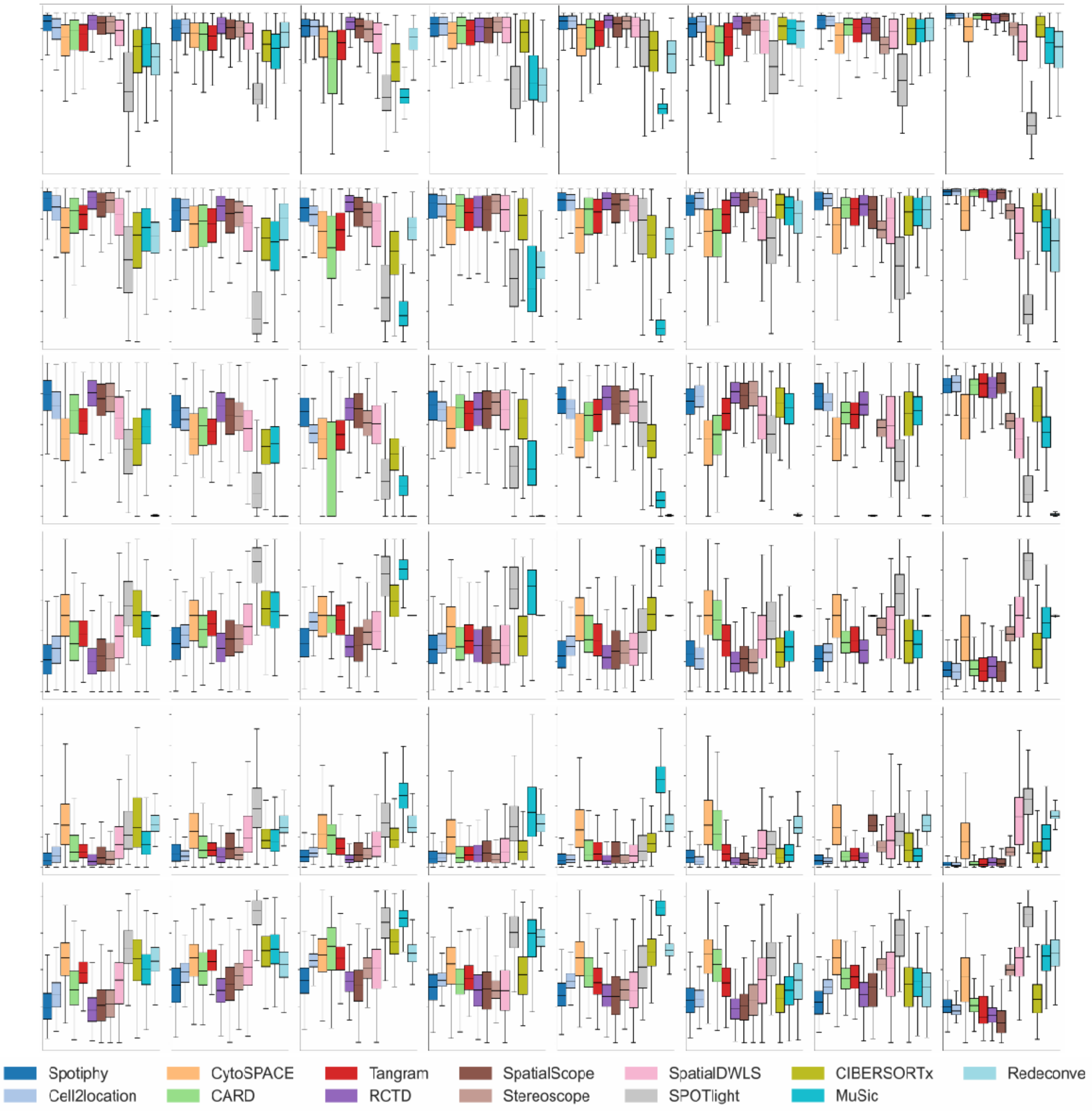

Supplementary Figure 11. The performance benchmarking using additional ST datasets. a-f, Box plots for correlation (**a**), cosine similarity (**b**), fraction of cells correctly mapped (**c**), absolute error (**d**), square error (**e**), and JSD (**f**) of the cell-type proportions for each transcriptomic spot generated by each method. Boxplots are generated in the same manner as described in Figure 2. Each dataset includes 1000 spots.

Supplementary Figure 12

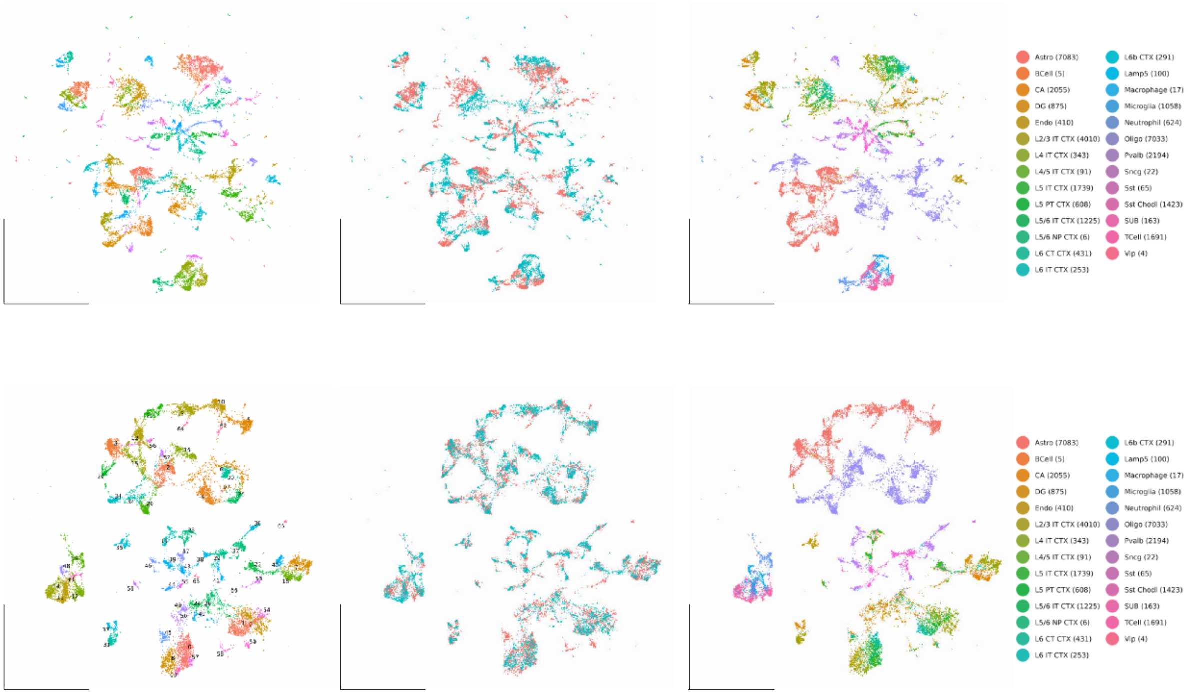

Supplementary Figure 12. UMAP projection of 33,819 cells from iscRNA data using Seurat without (a) and with Harmony (b) integration method.

Supplementary Figure 13

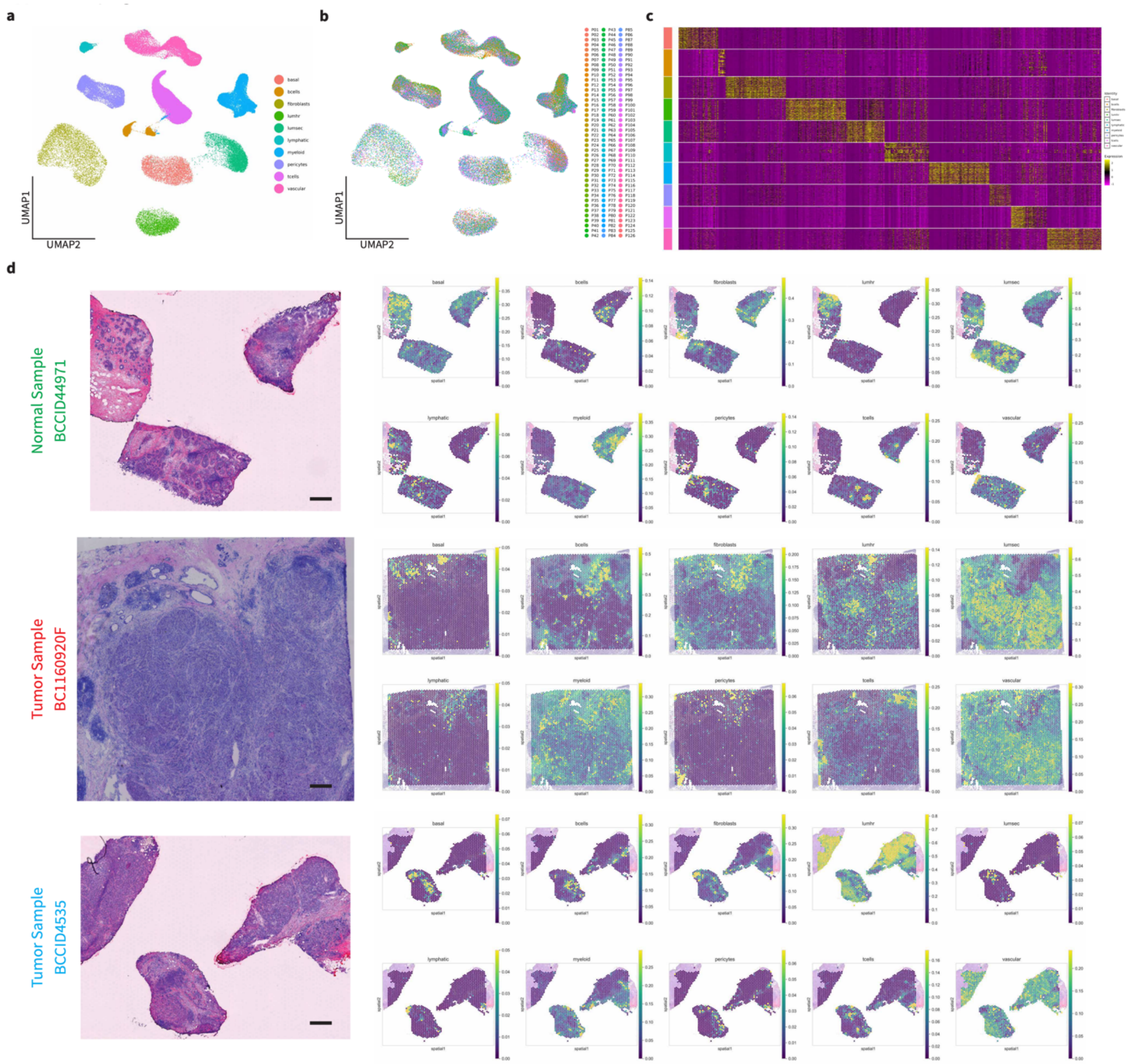

Supplementary Figure 13. Spotiphy provides cell-type proportions of human breast samples. a-b, UMAP projection of cells from scRNA-seq data used as human breast reference. Cells are labeled according to cell type (**a**) and sample-of-origin (**b**). **c,** Heatmap of expression of marker genes among 10 cell types. **d,** Heatmaps depicting the proportion of 10 cell types generated by Spotiphy across the histological images of three human breast Visium samples. Visium data includes one biological replicate for each tumor sample. Scale bars: 500 μm.

Supplementary Figure 14

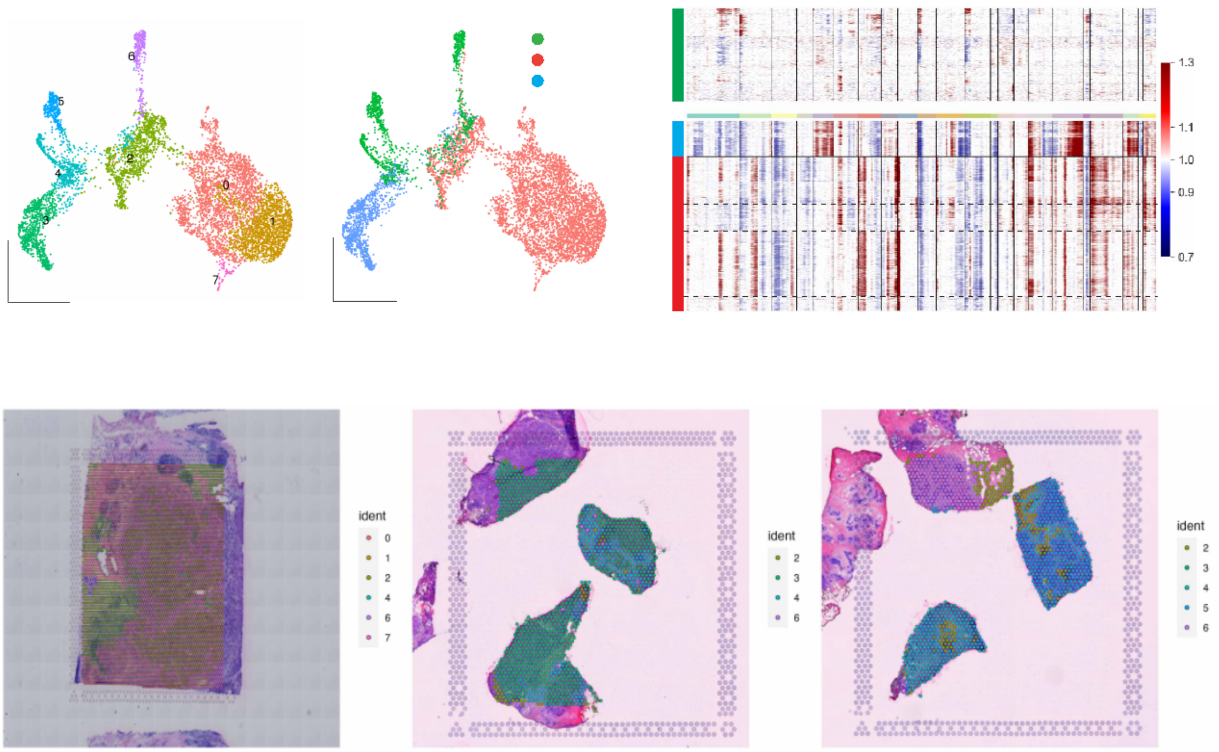

Supplementary Figure 14. a-b, UMAP projection of 7,183 spots from three human breast samples. **c,** inferCNV results using spot-level ST data. **d,** Transcriptomic spots from the Visium data are color-coded according to their spots' clusters.

Supplementary Figure 15

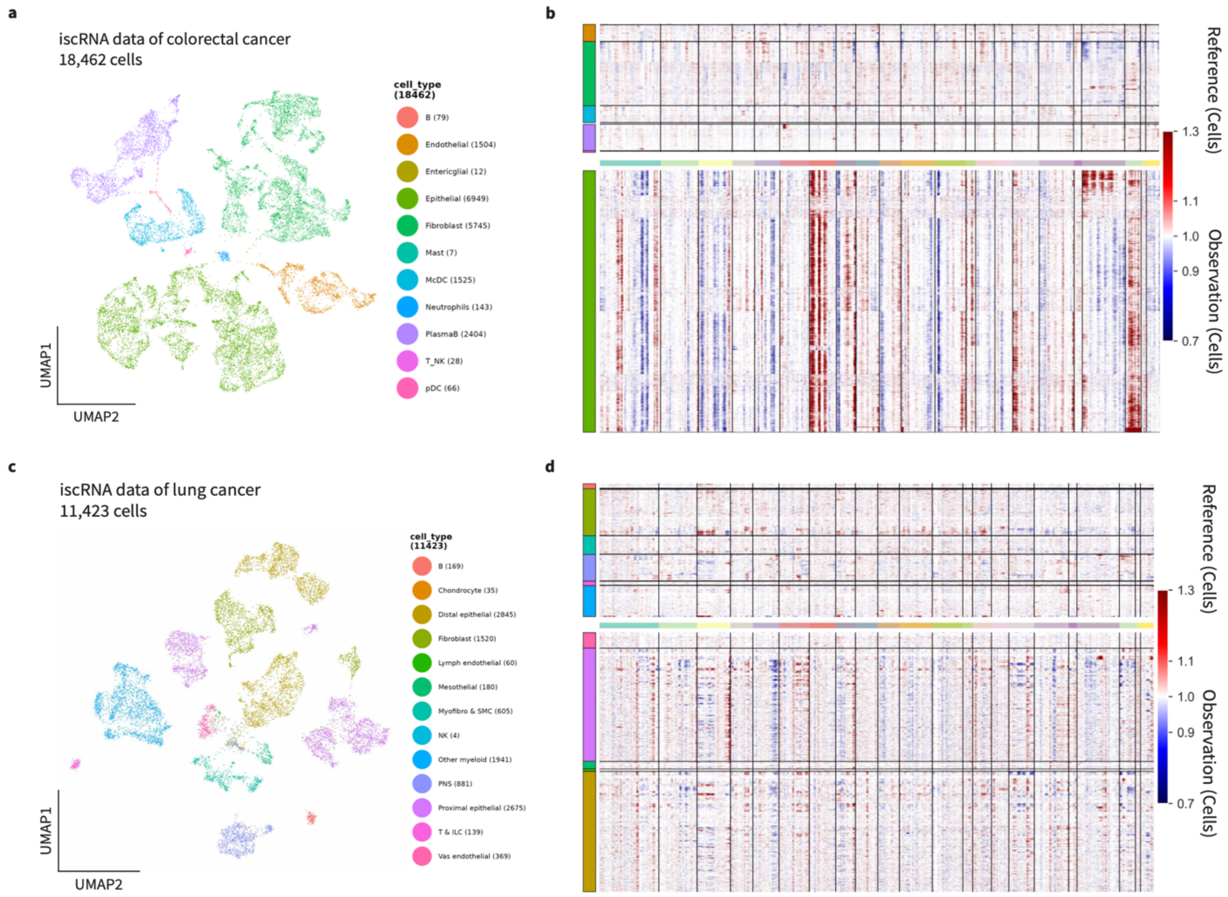

Supplementary Figure 15. a-b, Human colorectal cancer sample. **a,** UMAP projection of 18,462 cells of iscRNA data. **b,** inferCNV results using iscRNA data. **c-d,** Human lung cancer sample. **c,** UMAP projection of 11,423 cells of iscRNA data. **d,** inferCNV results using iscRNA data.

Supplementary Figure 16

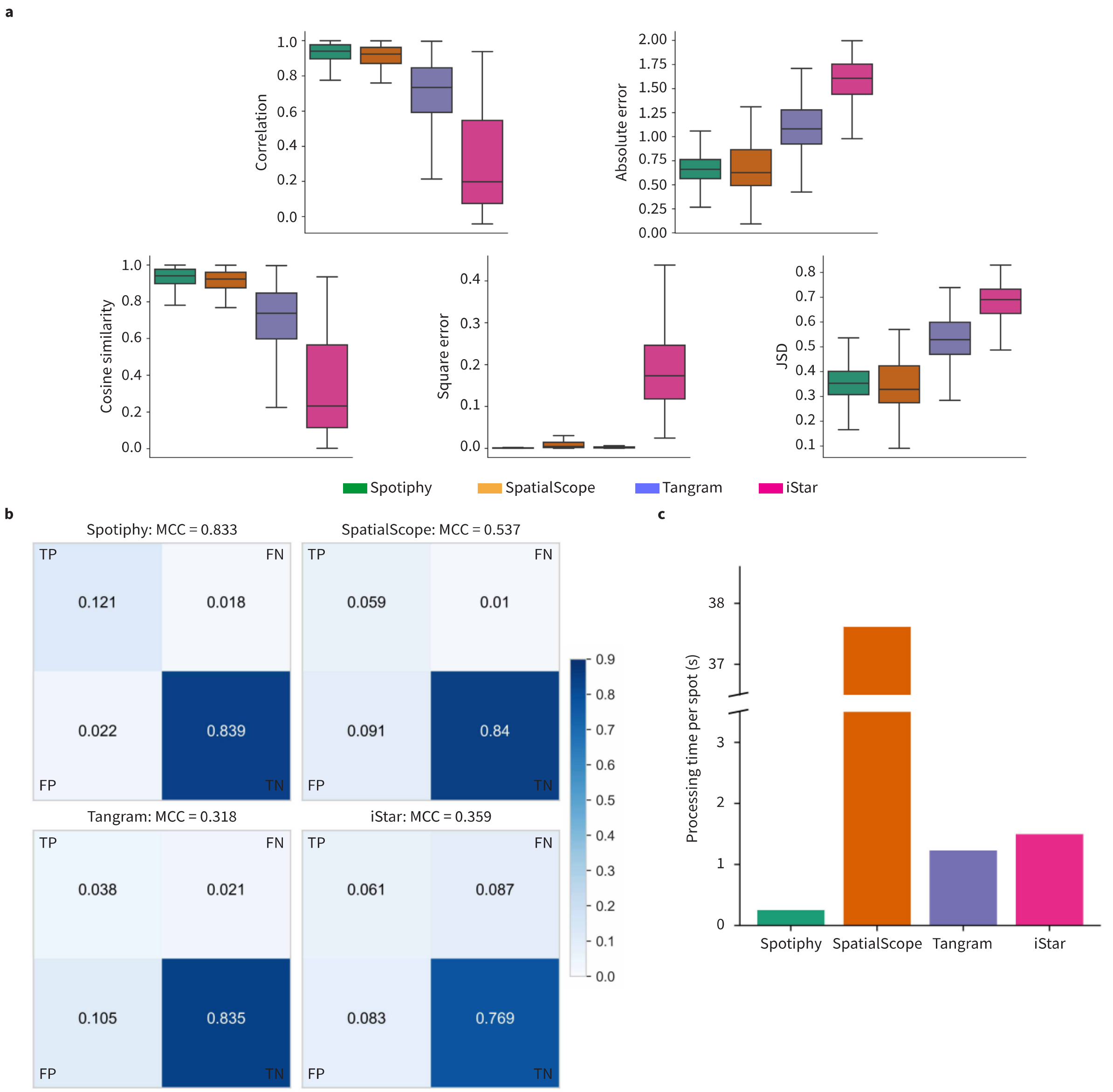

Supplementary Figure 16. Decomposition Evaluation using simulated Visium data of mouse brain. a, Box plots represents the correlation, absolute error, cosine similarity, square error, and JSD of the cell-type-level expression profiles for each transcriptomic spot generated by each method. Boxplots are generated in the same manner as described in Figure 2. Each platform includes 27 cell-type of AD sample. **b,** Confusion matrix for each method for Matthew's correlation coefficient (MCC) calculation. TP: true positive, FP: false positive, FN: false negative, TN: true negative. **c,** Decomposition processing time per spot.

Supplementary Figure 17

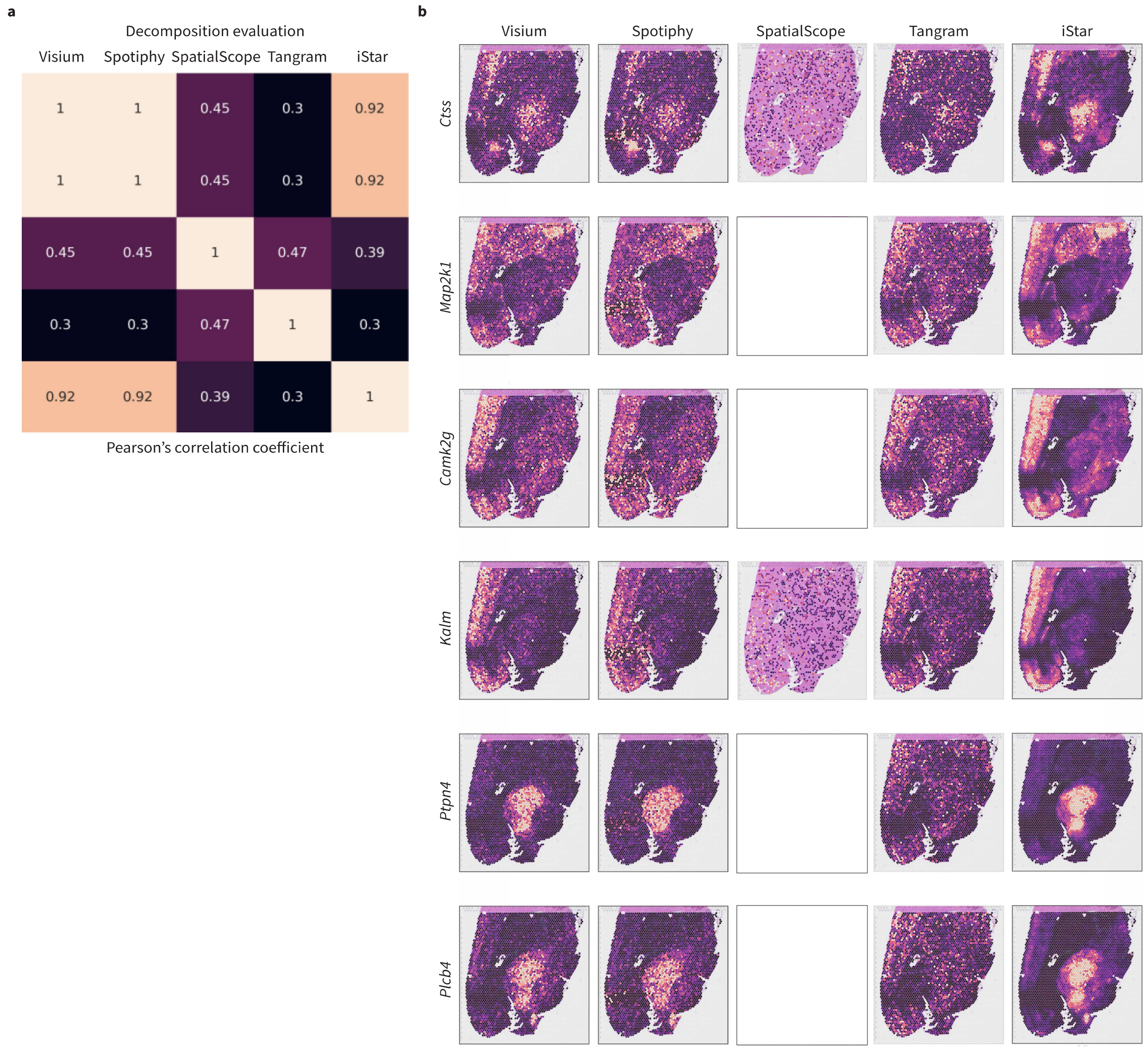

Supplementary Figure 17. Decomposition Evaluation using real Visium data of mouse brain. a, Pearson correlation coefficient heatmap of cell-type-level expression profiles generated by all methods selected for benchmarking. **b,** Selected SVGs distribution patterns across the histological section of AD mouse sample.

Supplementary Figure 18

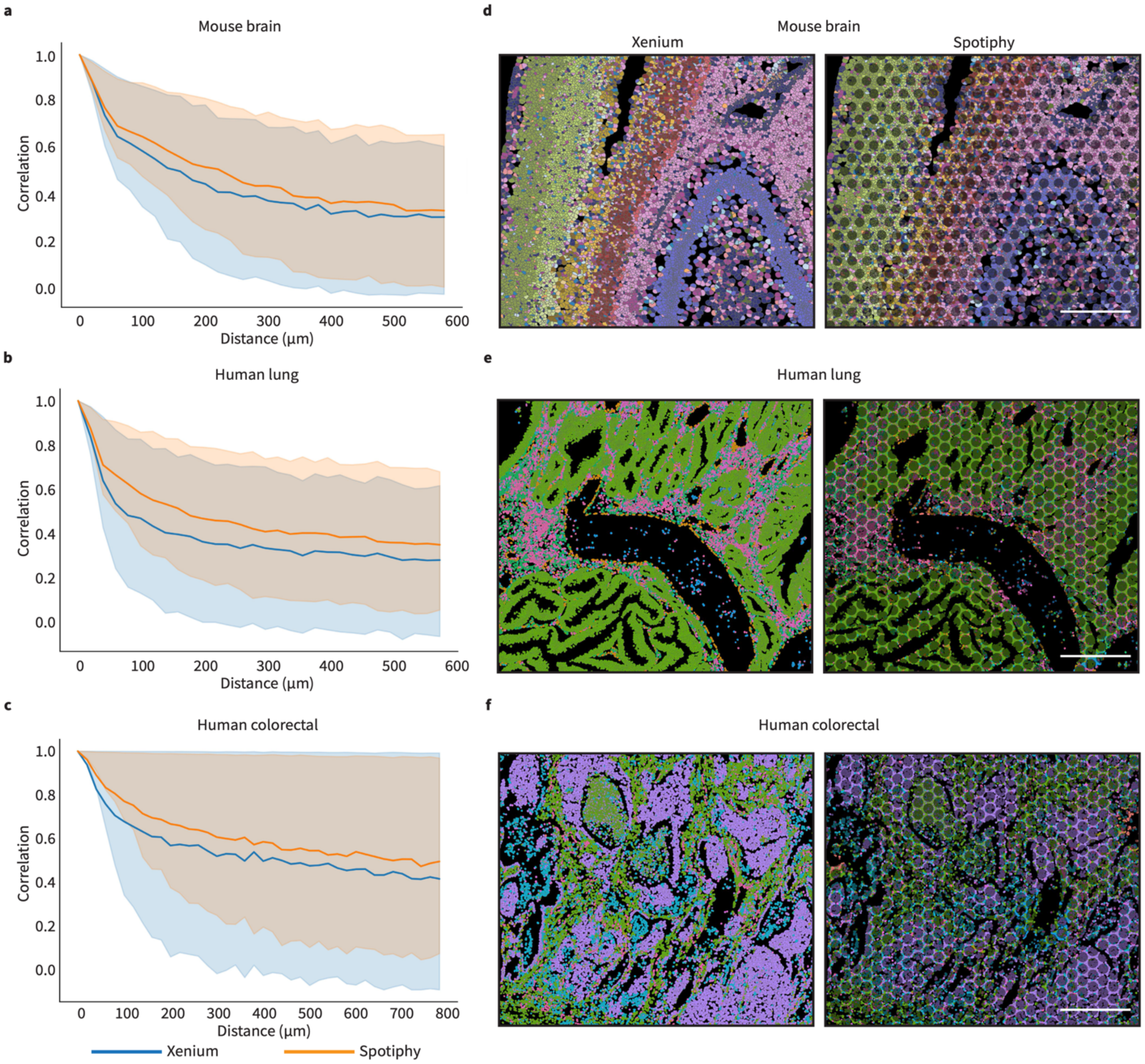

Supplementary Figure 18. Imputation evaluations using Xenium datasets. a-c, The correlation between distance and cellular proportion for 50-μm spots in mouse brain (**a**), human lung cancer (**b**), human colorectal (**c**) tissues. The error bands represent the standard deviations for each data point. **d-f,** Comparison between Xenium (the ground truth, left panel) and Spotiphy's imputation results (right panel) based on in-spot data of mouse brain (**d**), human lung cancer (**e**), human colorectal (**f**). Shadows in right panels represent spot location. Scale bar: 500 μm.

Supplementary Figure 19

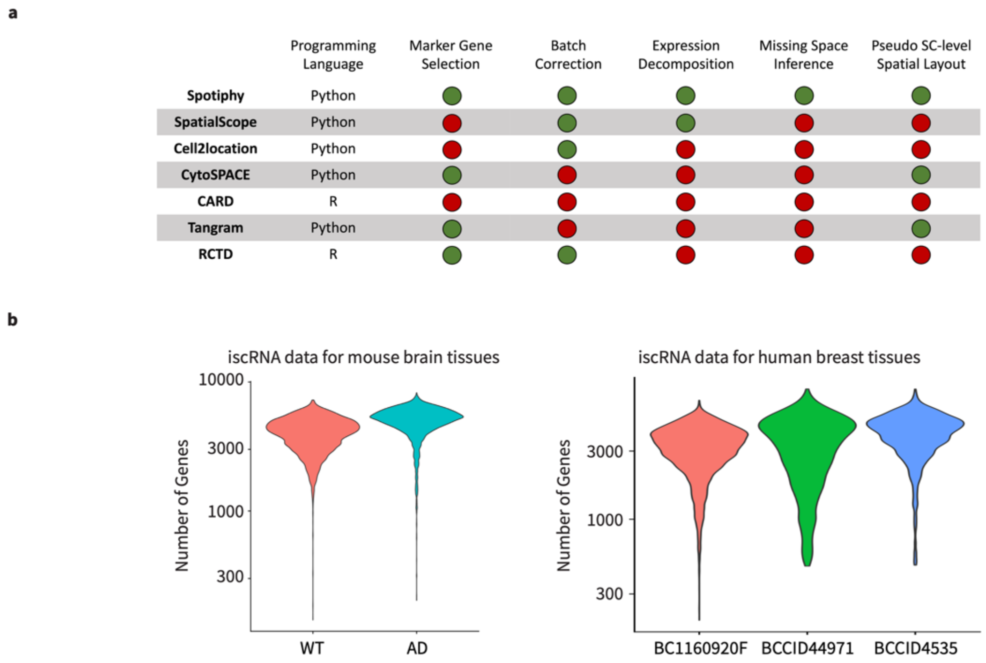

Supplementary Figure 19. Summary of benchmarking methods with Spotiphy. a, Major features of all methods benchmarked with Spotiphy in this study. **b,** Gene coverage of iscRNA data generated from Visium datasets used in this study. Left panel represents WT and AD mouse brain samples. Right panel represents three human breast samples.

Supplementary Figure 20

a

Pseudo single-cell-resolved whole-transcriptome image with cell annotation of WT mouse brain sample

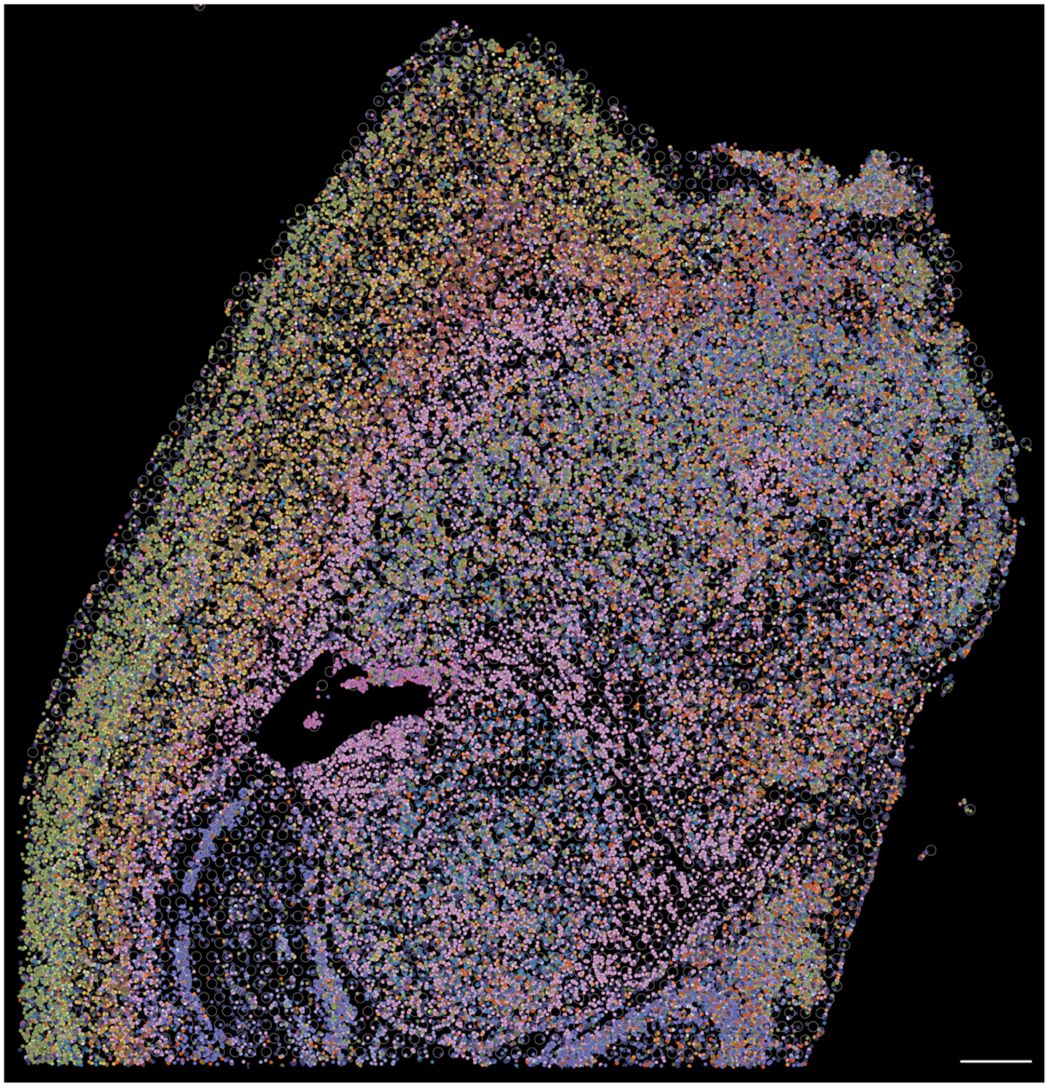

b

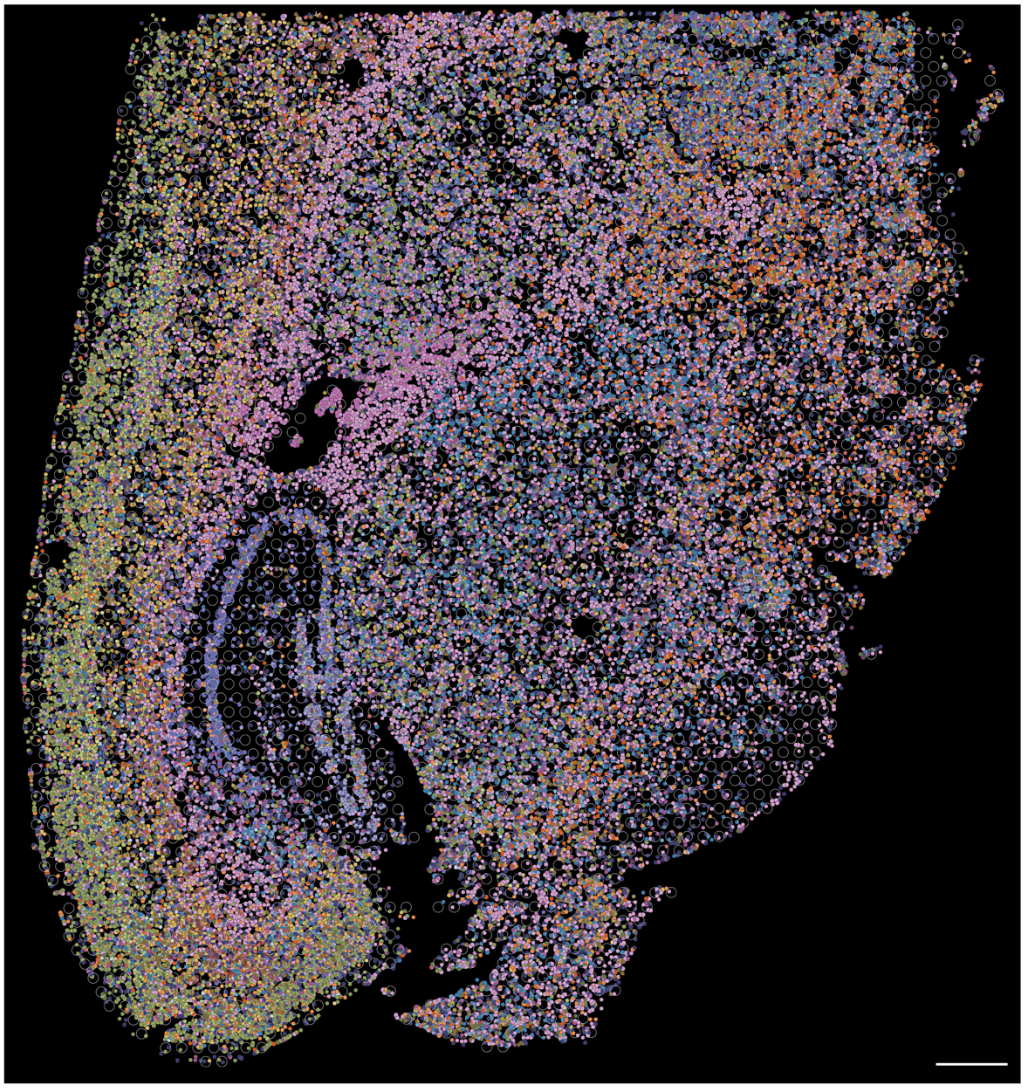

Supplementary Figure 20. Pseudo single-cell-resolved whole-transcriptome images with cell annotation of WT mouse brain sample (a) and of AD mouse brain sample (b). Color legend in **Fig. 6**. Scale bar: 500 μm.

Supplementary Methods

1 Spotiphy pipeline

Spotiphy is a unified pipeline that integrates scRNA-seq data, spatial transcriptomics (ST) data, and high-resolution histology images to enhance our understanding of complex biological systems. The core idea of Spotiphy is to effectively model the posterior distribution of spatial transcriptomics considering factors such as the single-cell reference, cell type proportions, and batch effects. With the generative modeling, Spotiphy primarily performs three tasks: 1. estimating the proportion of cell types at each capture area (e.g., circular spots) of the tissue; 2. generating inferred single-cell RNA expression matrix (iscRNA data); 3. generating pseudo single-cell resolution images. In this section, we present the workflow and detailed mathematics behind the pipeline. The structure of this section is illustrated in **Supplementary Information Fig. 1**.

The most commonly used notations in this section are briefly introduced as follows. We use bold uppercase letters to denote matrices and sets, bold lowercase letters to denote vectors, and unbolded letters to denote scalars. Besides, for a matrix $\boldsymbol{M}$, we use the corresponding lower letter notations $m_{i,j}$ and $\boldsymbol{m}_i$ to denote its ij-th entry and i-th row. We let $\boldsymbol{X} \in \mathbb{R}^{S\times G}$ denote the spatial expression count matrix after counts per million (CPM) normalization, where S is the number of capture areas (locations) and G is the number of gene types. Thus, $x_{s,g}$ represents the normalized expression of gene g at location s. We let $\boldsymbol{Y} \in \mathbb{R}^{C\times G}$ denote the normalized scRNA expression matrix, where C is the number of cells. In addition, to fully leverage matrix $\boldsymbol{Y}$, the cell types of all cells need to be annotated. We let $\tau(c) \in \{1, 2, \cdots, T\}$ denote the type of cell c, where T is the number of cell types.

1.1 Marker gene selection

ST data and scRNA-seq data typically detect over 10,000 pre-designed genes. However, not all of these genes are informative in differentiating cell types and estimating cell type proportions. For example, some genes may not be differentially expressed across different cell types, while others may have low expression levels that make them difficult to be detected reliably. Therefore, it is

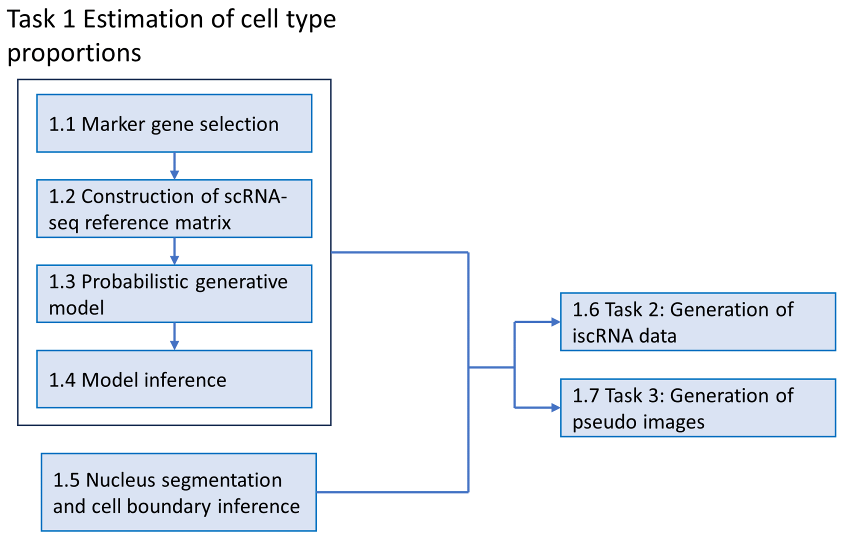

Supplementary Information Figure 1. Structure of supplementary methods.

important to identify a subset of marker genes that are informative in our analysis to enhance both the speed and accuracy of the estimation. Similar to the literature, the marker genes in Spotiphy are defined as the genes that exhibit significantly higher expression levels in one specific cell type compared to all other cell types.

In the literature, marker genes are usually selected based on one-vs-rest comparisons. Specifically, to determine whether a gene g is a marker gene for cell type t, statistical tests (or comparisons) are performed based on two populations: 1. the expression of gene g in type t cells, and 2. the expression of gene g in cells of all other types. However, one issue with this approach is that it involves aggregating the expression of gene g across all other types, which may lead to unreliable

results. For example, we let $\mu_{t,g}$ denote the average expression of gene g in type t cells, and assume that $\mu_{t,g} < \mu_{t',g}$. Thus, gene g has a higher expression level in type t' cells and should not be selected as the marker gene of cell type t. Nevertheless, after aggregating the expression of gene g in all cells that do not belong to type t, the mean of the first population can be significantly higher than that of the second population. In this case, the one-vs-rest approach may wrongly select gene g as the marker gene of cell type t. Another issue of the one-vs-rest approach is that the comparison results are influenced by the number of cells belonging to each cell type in the scRNA-seq data. For example, if 98% of the cells in the scRNA-seq data belong to cell type t, then the marker genes of cell type t' can merely distinguish cell types t and t'.

To address these issues, we propose a new method to select marker genes, which is based on pairwise z-test, pairwise fold changes, and coverage rates. Specifically, we let $\boldsymbol{A}_t = \{c = 1, 2, \cdots, C | \tau(c) = T\}$ denote the set of cell indices that belongs to cell type t. Then the mean and variance of gene g expression in type t cells are estimated as

$$\hat{\mu}_{t,g} = \frac{\sum_{c \in \boldsymbol{A}_t} y_{t,g}}{|\boldsymbol{A}_t|}, \qquad \hat{\sigma}_{t,g} = \sqrt{\frac{\sum_{c \in \boldsymbol{A}_t} \left(y_{t,g} - \hat{\mu}_{t,g}\right)^2}{|\boldsymbol{A}_t| - 1}}, \tag{1}$$

where $|\boldsymbol{A}_t|$ is the cardinality of set $\boldsymbol{A}_t$. Thus, to determine whether $\mu_{t,g} > \mu_{t',g}$, we conducte z-test with null hypothesis $H_0\colon \mu_{t,g} \leq \mu_{t',g}$ and alternative hypothesis $H_1\colon \mu_{t,g} > \mu_{t',g}$. The test statistic is derived as

$$z_{g,t,t'} = \frac{\hat{\mu}_{t,g} - \hat{\mu}_{t',g}}{\sqrt{\frac{\left(\hat{\sigma}_{t,g}\right)^2}{|\boldsymbol{A}_t|} + \frac{\left(\hat{\sigma}_{t',g}\right)^2}{|\boldsymbol{A}_{t'}|}}}. \tag{2}$$

We let $\lambda_{g,t,t'}$ denote the p-value of this test. Besides, we let $\boldsymbol{F}_{g,t} = \{\hat{\mu}_{t,g}/\hat{\mu}_{t',g} | t' \neq t\}$ denote the set of fold changes for gene g when comparing gene expressions in cell type t with each other cell type. We let $f_{g,t}(v)$ denote the v-th sample quantile for the values in set $\boldsymbol{F}_{g,t}$. Furthermore, we let $w_{t,g} = \frac{\sum_{c \in \boldsymbol{A}_t} I(y_{t,g} > 0)}{|\boldsymbol{A}_t|}$ be the coverage rate of gene g in type t cells, where I is the indicator function that takes the value 1 when the specified condition is satisfied and 0 otherwise. With these preparations, gene g is selected as a candidate of type t marker genes when the following three conditions are satisfied:

- $w_{t,g} > l_{\text{cover}}$,
- $\max\{\lambda_{g,t,t'} | t' \neq t\} < l_{\lambda}$,
- $f_{g,t}(v) > l_{\text{fold}}$.

The first condition requires that the gene g be expressed in a certain proportion of the type t cells. The second condition requires all pairwise statistics related to $\mu_{t,g}$ are significant. Finally, the last condition guarantees that the gene g exhibits higher expression in type t cells compared to cells of other types, in terms of fold change. Note that for the fold change requirement, we incorporate the parameter v for calculating the quantiles instead of using the minimum fold change from set $\boldsymbol{F}_{g,t}$. This is because setting a requirement for the minimum fold change to exceed a certain threshold can be overly stringent, particularly when some cell types are very similar, such as neurons at different layers of the cerebral cortex.

In these conditions, l_{fold}, l_{λ}, and l_{cover} are predetermined thresholds and v is the predetermined quantile level. Besides, if cell type t has more than n_{select} candidate genes, we rank them based

on the fold change quantile $f_{g,t}(v)$, and only select the top n_{select} genes. In this way, the selected marker gene will not be dominated by any single cell type. We repeat this process for all cell types and aggregate the selected marker genes. Based on our preliminary results, the proposed method works well when we select $l_{\text{fold}} = 1.5$, $l_{\lambda} = 0.1$, $l_{\text{cover}} = 60\%$, $v = 0.15$, and $n_{\text{select}} = 50$.

By only keeping the marker genes in matrices $\boldsymbol{X}$ and $\boldsymbol{Y}$, we obtain matrices $\boldsymbol{X}^{(\text{m})} \in \mathbb{R}^{S\times G_{\text{m}}}$ and $\boldsymbol{Y}^{(\text{m})} \in \mathbb{R}^{C\times G_{\text{m}}}$, where G_{m} is the total number of selected marker genes, and the superscript and subscript "m" means that only the marker genes are considered.

1.2 Construction of scRNA-seq reference matrix

We let $\boldsymbol{\Phi}^{(\text{m})} \in \mathbb{R}^{T\times G_{\text{m}}}$ be the scRNA-seq reference matrix constructed based on $\boldsymbol{Y}^{(\text{m})}$, where $\varphi_{t,g}^{(\text{m})}$ represents the average proportion of gene g in expression of type t cells. Thus, we have $\sum_{g=1}^{G_{\text{m}}} \varphi_{t,g}^{(\text{m})} = 1$ for $t = 1, 2, \cdots, T$. Similar to BayesPrism[69], we assumed that the expression of each cell follows the multinomial distribution:

$$\boldsymbol{y}_c^{(\text{m})} \sim \text{multinomial}(m_c, \boldsymbol{\varphi}_{\tau(c)}^{(\text{m})}), \tag{3}$$

where $\boldsymbol{y}_c^{(\text{m})}$ is the c-th row of matrix $\boldsymbol{Y}^{(\text{m})}$, $m_c = \sum_{g=1}^{G_{\text{m}}} y_{c,g}^{(\text{m})}$ is the total gene count of cell type c, $\tau(c)$ is the cell type index of cell c, and $\boldsymbol{\varphi}_{\tau(c)}^{(\text{m})}$ is the $\tau(c)$-th row of reference matrix $\boldsymbol{\Phi}^{(\text{m})}$. Therefore, the likelihood of $\boldsymbol{\varphi}_t^{(\text{m})}$ is expressed as

$$L\left(\boldsymbol{\varphi}_t^{(\text{m})}\right) \propto \prod_{c\in \boldsymbol{A}_t} \prod_{g=1}^{G_{\text{m}}} \left(\varphi_{t,g}^{(\text{m})}\right)^{y_{c,g}^{(\text{m})}}, \tag{4}$$

By maximizing the likelihood, the entries of reference matrix $\boldsymbol{\Phi}^{(\text{m})}$ is estimated as

$$\hat{\varphi}_{t,g}^{(\mathrm{m})} = \frac{\sum_{c \in \boldsymbol{A}_t} y_{c,g}^{(\mathrm{m})}}{\sum_{c \in \boldsymbol{A}_t} \sum_{g'=1}^{G_\mathrm{m}} y_{c,g'}^{(\mathrm{m})}}. \tag{5}$$

An issue with this approach is that CPM normalization may cause the value of $y_{c,g}$ to be a non-integer, making the distribution assumption (3) invalid. To address this issue, we can round the matrix $\boldsymbol{Y}$ such that all the entries are integers. However, since the calculation of $\hat{\varphi}_{t,g}$ in equation (5) does not require $y_{c,g}$ to be an integer, rounding the matrix $\boldsymbol{Y}$ only has a minor influence on the estimated value of $\hat{\varphi}_{t,g}$, and thus is not necessary. In the following step, we will discard the scRNA expression matrix $\boldsymbol{Y}^{(\mathrm{m})}$ and only used the estimated single-cell reference matrix $\widehat{\boldsymbol{\Phi}}^{(\mathrm{m})}$ in our analysis.

1.3 Probabilistic generative model

To infer the cell type proportions and decompose expression at the single-cell level, we leverage a probabilistic generative model where both the spatial expression and the scRNA reference are integrated. The model is depicted in **Supplementary Information Fig. 2**, where the gray circles represent the known variables or observations, the white circles represent the unknown parameters that need to be estimated, and the squares represent the hyperparameters we choose before the inference.

Specifically, we let $\boldsymbol{Q} \in \mathbb{R}^{S \times T}$ be the proportion matrix of contributed genes, where $q_{s,t}$ denotes the proportion of genes at location s that are contributed by type t cells. Thus, $\sum_{t=1}^{T} q_{s,t} = 1$ for $s = 1, 2, \cdots, S$. It is worth noting that $q_{s,t}$ does not equal to $p_{s,t}$, which is the proportion of type t cells at location s. This is because when only considering the G_m marker genes, cells belongs to

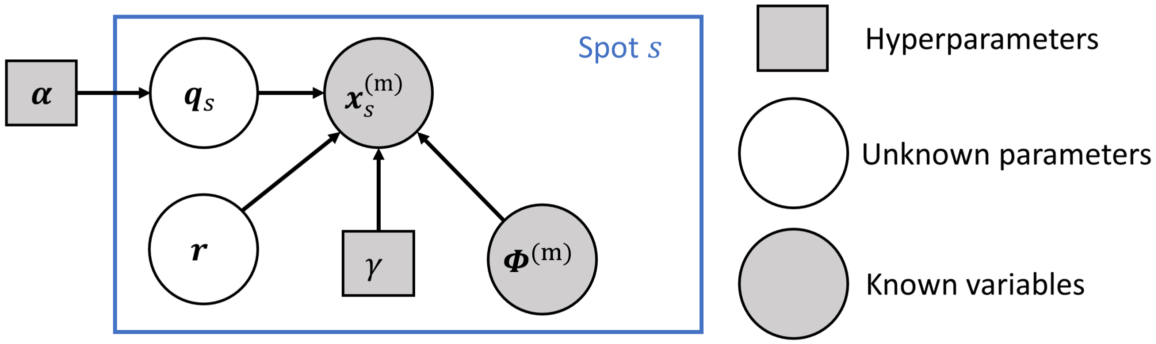

Supplementary Information Figure 2. Graphical illustration of the probabilistic generative model.

different cell type have different average total count in spatial tissues. For example, suppose $p_{s,t_1} = p_{s,t_2} = 0.5$, which means the two cell types have the same number of cells at location s. If type t_1 cells have higher total expression than type t_2 cells at location s, then we have $q_{s,t_1} > 0.5 > q_{s,t_2}$. For location $s = 1, 2, \cdots, S$, we let the prior distribution of $\boldsymbol{q}_s = [q_{s,1}, q_{s,2}, \cdots, q_{s,T}]$ be

$$\boldsymbol{q}_s \sim \text{Dirichlet}(\boldsymbol{\alpha}), \tag{6}$$

where $\boldsymbol{\alpha} = [3, 3, \cdots, 3]$ is the hyperparameter that indicates a weak prior. Note that we have tested other values of $\boldsymbol{\alpha}$, and the results are very similar, except when the prior is highly informative.

Recall that $\varphi_{t,g}^{(\mathrm{m})}$ represents the average proportion of gene g in type t cells. Thus, if there is no batch effect, the proportion of gene g at location s can be derived as

$$\rho_{s,g} = \sum_{t=1}^{T} q_{s,t} \cdot \varphi_{t,g}^{(\mathrm{m})}. \tag{7}$$

The intuition behind equation (7) is that when one gene at location s is randomly selected, there is a probability $q_{s,t}$ that this gene is contributed by cells of type t. When such gene is contributed by cells of type t, the probability that it belongs to gene type g is $\varphi_{t,g}^{(\mathrm{m})}$.

However, one issue of equation (7) is that the batch effects between the scRNA-seq expression and the spatial expression are not considered. To quantify the batch effects, we introduce batch effect parameters $\boldsymbol{r} = \{r_1, r_2, \cdots, r_G\}$, where

$$r_g \sim \text{Unif}(0, 1), \quad \text{for } g = 1, 2, \cdots, G_m. \tag{8}$$

Then the proportion of gene g at location s is adjusted as

$$\tilde{\rho}_{s,g} = \frac{\rho_{s,g} \cdot 2^{\gamma r_g}}{\sum_{g'=1}^{G_\text{m}} \rho_{s,g'} \cdot 2^{\gamma r_{g'}}}. \tag{9}$$

where $\gamma = 2$ is a hyperparameter. From equation (9), we deduce the following two findings. First, when r_g is large, gene g has higher proportion in spatial expression due to the batch effect. Second, the hyperparameter γ controls the dispersion of the batch effects among the genes. In other words, in equation (9), the highest possible weight of $\rho_{s,g}$ is 2^γ and the lowest possible weight of $\rho_{s,g}$ is 1. According to our experiments, γ should not be larger than 4.

With the preparation above, the conditional distribution of $\boldsymbol{x}_s^{(\text{m})}$ is modeled as the multinomial distribution:

$$\boldsymbol{x}_s^{(\text{m})} | \boldsymbol{Q}, \boldsymbol{r}, \boldsymbol{\Phi}^{(\text{m})}, \gamma \sim \text{Multinomial}\left(m'_s, \left[\tilde{\rho}_{s,1}, \tilde{\rho}_{s,2}, \cdots, \tilde{\rho}_{s,G_\text{m}}\right]\right), \tag{10}$$

where $m'_s = \sum_{g=1}^{G_\text{m}} x_{s,g}^{(\text{m})}$ is the total gene count at location s.

This generative model describes the generation of spatial expression in a probabilistic manner based on relevant parameters. Our model offers two unique advantages compared to existing probabilistic generative models used in cell type deconvolution. Firstly, it uniquely considers how spatial expression at location s can be decomposed for each cell type. This feature enables the generation of iscRNA data in Subsection 1.6. Secondly, in comparison to other methods, our model

is more straightforward, with significantly fewer tuning parameters. This simplicity contributes to reduced computational time.

1.4 Model inference

In the probabilistic generative model, the unknown parameters are the proportion matrix $\boldsymbol{Q}$ and the batch effect parameter $\boldsymbol{r}$. To estimate these parameters through the model, we need to conduct Bayesian inference and calculate the posterior distributions $\mathrm{P}(\boldsymbol{Q}|\boldsymbol{X}^{(\mathrm{m})},\boldsymbol{\Phi}^{(\mathrm{m})},\gamma)$ and $\mathrm{P}(\boldsymbol{r}|\boldsymbol{X}^{(\mathrm{m})},\boldsymbol{\Phi}^{(\mathrm{m})},\gamma)$. However, calculating the exact posterior distributions is intractable due to the inherent complexity of the model. Therefore, we leverage variational inference to approximate the true posterior distributions.

Specifically, we assume that

$$\mathrm{P}(\boldsymbol{Q},\boldsymbol{r}|\boldsymbol{X}^{(\mathrm{m})},\boldsymbol{\Phi}^{(\mathrm{m})},\gamma) \approx \prod_{s=1}^{S} Q(\boldsymbol{q}_s|\boldsymbol{\alpha}_s) \prod_{g=1}^{G_m} Q\left(r_s\middle|\mu_g^{(r)},\sigma_g^{(r)}\right), \tag{11}$$

where $Q(\boldsymbol{q}_s|\boldsymbol{\alpha}_s)$ is the probability density function (PDF) of distribution $\mathrm{Dirichlet}(\boldsymbol{\alpha}_s)$, and $Q\left(r_s\middle|\mu_g^{(r)},\sigma_g^{(r)}\right)$ is the PDF of distribution $N(\mu_g^{(r)},\sigma_g^{(r)})$. By applying variational inference, we optimize the parameters $\boldsymbol{\alpha}_s$, $\mu_g^{(r)}$, and $\sigma_g^{(r)}$ to maximize the Evidence Lower Bound (ELBO). This maximization is equivalent to minimizing the Kullback-Leibler (KL) divergence between distributions $\mathrm{P}(\boldsymbol{Q},\boldsymbol{r}|\boldsymbol{X}^{(\mathrm{m})},\boldsymbol{\Phi}^{(\mathrm{m})},\gamma)$ and $\prod_{s=1}^{S} Q(\boldsymbol{q}_s|\boldsymbol{\alpha}_s) \prod_{g=1}^{G_m} Q\left(r_s\middle|\mu_g^{(r)},\sigma_g^{(r)}\right)$. The variational inference is implemented using the Python package Pyro[70], which supports GPU acceleration.

By slightly abusing the notation, we let $\boldsymbol{\alpha}_s$, $\mu_g^{(r)}$, and $\sigma_g^{(r)}$ denote the optimized parameters. Then the parameters $\boldsymbol{Q}$ and $\boldsymbol{r}$ are estimated by the posterior mean:

$$\hat{q}_{s,t} = \frac{\alpha_{s,t}}{\sum_{t'=1}^{T} \alpha_{s,t'}}, \tag{12}$$

$$\hat{r}_g = \mu_g^{(r)}. \tag{13}$$

Recall that $q_{s,t}$ represents the proportion of genes contributed by type t cells, rather than the proportion of type t cell in terms of absolute cell numbers. To get the proportion of type t cells at location s, we let h_t be the average total gene count of type t cells when only the marker genes are considered, which is calculated as $h_t = \frac{1}{|\boldsymbol{A}_t|} \sum_{c \in \boldsymbol{A}_t} \sum_{g=1}^{G_m} y_{c,g}^{(\mathrm{m})}$. Finally, the following transformation are used to estimate the proportion of type t cells at location s:

$$\hat{p}_{s,t} = \frac{\hat{q}_{s,t}/h_t}{\sum_{t'}^{T} (\hat{q}_{s,t'}/h_{t'})}. \tag{14}$$

1.5 Nucleus segmentation and cell boundary inference

High-resolution Hematoxylin and Eosin (H&E) staining images provide us with information on nucleus locations, as hematoxylin stains the nuclei purple and eosin stains the surrounding cytoplasm pink. In Spotiphy, we utilize high-resolution H&E staining images for two primary purposes. Firstly, they allow us to determine the location of nuclei and infer cell boundaries based on the image data. These outcomes are necessary for generating pseudo single-cell resolution images. Secondly, using the identified nuclei locations, we can calculate the number of cells in each capture area and decompose the expression of each capture area to the single-cell level, thereby generating iscRNA data. Although high-resolution images are recommended, they are not necessary for generating iscRNA data. Further details on alternative options are described in Subsection 1.6.

To extract useful information from H&E stained images, segmentation is required to separate the pixels of nuclei from the background. In Spotiphy pipeline, we adopted the pretrained deep learning model from Stardist[71,72] to segment the nuclei. After the segmentation, the background pixels are labeled as 0 and the pixels of each nucleus are labeled as the index of the nucleus. With the segmentation results, we assume that a cell is located at position s if the center of the cell's nucleus falls within the capture area.

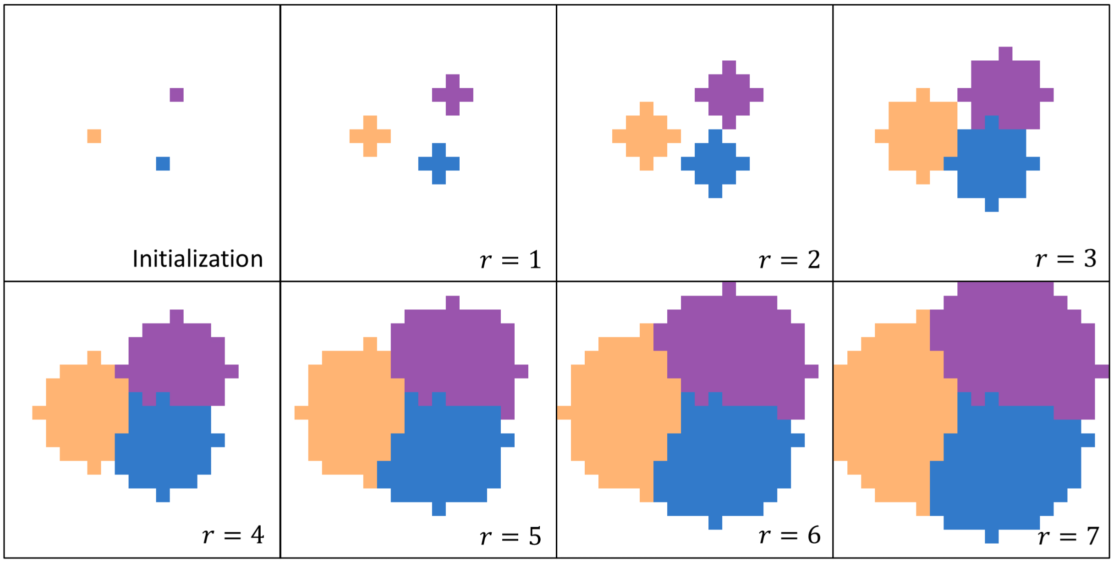

Supplementary Information Figure 3. Illustration of the cell expansion when $r \leq 7$.

We then let N_s denote the number of cells at location s. To infer the cell boundaries based on the segmentation result, we let dist_{max} denote the maximum distance from a point on the cell boundary to the corresponding nuclei center and let Area_{max} denote the maximum area of a cell. Both quantities are measured by the image pixels. Initially, we set $\omega = 1$ and assume each cell only occupies one pixel, which is the center of the nuclei. In each iteration, we expand the pixels of each cell. Specifically, we increase ω by $\Delta\omega$. Then, we iterate over all cells, allowing each cell to occupy all the background pixels within a Euclidean distance of ω pixels from its nucleus center.

The expansion of a cell is halted when all surrounding pixels are occupied by other cells, or the constraints set by $\mathrm{dist}_{\mathrm{max}}$ and $\mathrm{Area}_{\mathrm{max}}$ restrict further expansion of the cell. The cell boundaries can then be determined as the pixels of cell that are sufficiently close to other cells or the background. The first seven iterations of this algorithm when we set $\Delta\omega = 1$ is illustrated in **Supplementary Information Fig. 3**.

1.6 Generation of iscRNA data

To generate iscRNA data for all cells in the tissue, we first generate iscRNA data for cells within the capture areas. Since the expressions of each area are known, the iscRNA data for these cells can be obtained by decomposing spatial expressions to the single-cell level. For cells outside the capture areas, obtaining their iscRNA data is more challenging and may introduces larger errors, since even aggregated expressions are unavailable. To this end, we provide an optional function in Spotiphy to impute the expression of cells outside the capture areas using the kernel smoothing method.

1.6.1 Decomposition of spatial expression

When a cell type does not exist at location s and we try to assign some spatial expression at location s to that cell type, the decomposition of spatial expression may have large errors. Thus, we first update the estimated cell type proportions before the decomposition based on the number of cells at each location. Specifically, we let N_s denote the number of cells at location s. In Spotiphy, we offer four options for N_s value.

First, when H&E staining image is available, N_s can be calculated based on nucleus segmentation result. Second, when the high-resolution image is not available, we can estimate the value of N_s based on the size of the raw counts at location s. Furthermore, we can also manually set the value according to domain knowledge. For $t = 1, 2, \cdots, T$, we let $n_{s,t}$ denote the number of type t cells at location s. Then the number of each cell type at location s can be determined by solving the following optimization problem:

$$\min \sum_{t=1}^{T} \left| \frac{n_{s,t}}{N_s} - p_{s,t} \right|, \tag{15}$$

$$\text{s.t.} \sum_{t=1}^{T} n_{s,t} = N_s. \tag{16}$$

This optimization problem can be easily solved using greedy algorithms. As a result, cell type proportions at location s are updated as $\tilde{p}_{s,t} = n_{s,t}/N_s$, for $t = 1, 2, \cdots, T$.

Finally, when the value of N_s cannot be calculated or estimated, we also provide the option to indicate that the value of N_s is missing. In this case, we define the threshold l_{p} and assume that if $p_{s,t} < l_{\mathrm{p}}$, there is no strong evidence to suggest the existence of cell type t at location s. Consequently, the proportion is updated as follows:

$$\tilde{p}_{s,t} = \begin{cases} 0, & p_{s,t} < l_{\mathrm{p}} \\ \dfrac{\sum_{t_1=1}^{T} p_{s,t_2} \cdot I(p_{s,t_1} \geq l_{\mathrm{p}})}{\sum_{t_2=1}^{T} I(p_{s,t_2} \geq l_{\mathrm{p}})}, & p_{s,t} \geq l_{\mathrm{p}} \end{cases}, \tag{17}$$

where $I(\cdot)$ is the indicator function. In default, the threshold l_{p} is set to 0.1. After updating the cell type proportions, we let $\widetilde{\boldsymbol{P}} \in \mathbb{R}^{S \times T}$ be the corresponding matrix where the st-th entry is $\tilde{p}_{s,t}$.

To make sure that the decomposed spatial expression can facilitate more downstream analysis, we aim to decompose the spatial expression of all G genes, rather than the G_m marker genes. Thus, we construct the single-cell reference matrix $\boldsymbol{\Phi} \in \mathbb{R}^{T\times G}$ using the full scRNA count matrix $\boldsymbol{Y}$ according to Subsection 1.2. In addition, we let $\boldsymbol{U} \in \mathbb{R}^{S\times G\times T}$ denote a 3-dimensional tensor, where $u_{s,t,g}$ is the expression of gene g in type t cells at location s. As a result, $\boldsymbol{u}_{s,g} = [u_{s,g,1}, u_{s,g,2}, \cdots, u_{s,g,T}]$ is the decomposition of spatial expression $x_{s,g}$, where we have $\sum_{t=1}^{T} u_{s,g,t} = x_{s,g}$. Note that we assume that when considering all G genes, the total expression of each cell at location s is identical. Therefore, when all genes are considered, the probability that a randomly picked gene at location s belongs to type t cells $\tilde{p}_{s,t}$.

By apply the probabilistic model again without considering the batch effect, we have

$$u_{s,g,t} \sim \text{Binomial}\left(\sum_{g=1}^{G} u_{s,g,t}, \tilde{p}_{s,t}\varphi_{t,g}\right), \tag{18}$$

and $[u_{s,g,t};\ g = 1, 2, \cdots, G;\ t = 1, 2, \cdots, T]$ follow the multinomial distribution. The intuition is that with probability $\tilde{p}_{s,t}\varphi_{t,g}$, a randomly selected gene at location s is a gene g contributed by a cell that belongs to type t. Finally, condition on $x_{s,g} = \sum_{t=1}^{T} u_{s,g,t}$, $\boldsymbol{u}_{s,g}$ also follows the multinomial distribution,

$$\boldsymbol{u}_{s,g} | x_{s,g} \sim \text{Multinomial}\left(x_{s,t}, [\omega_{s,g,1}, \omega_{s,g,2}, \cdots, \omega_{s,g,T}]\right), \tag{19}$$

where $\omega_{s,g,t} = \frac{\tilde{p}_{s,t}\cdot\varphi_{t,g}}{\sum_{t'=1}^{T} \tilde{p}_{s,t'}\cdot\varphi_{t',g}}$. Then $u_{s,t,g}$ can be estimated as the posterior mean: $\hat{u}_{s,t,g} = x_{s,t} \cdot \omega_{s,g,t}$. Note that when the proportion $\tilde{p}_{s,t}$ is 0, the decomposed spatial expression for type t cells at location s is always 0. Finally, the iscRNA data for cells inside the capture areas are obtained by reshaping the tensor $\boldsymbol{U}$.

1.6.2 Imputation of expression for cells in non-capture areas

Spotiphy includes an optional function to impute the expression for cells in non-capture areas through kernel smoothing. Specifically, for a cell c belonging to type t in a non-capture area, we consider all type t cells within the captured areas and derive weights for these cells based on their distance from cell c. Consequently, the expression of cell c is obtained as the weighted sum of the expressions of type t cells in the capture areas.

1.7 Generation of pseudo images

Recall that we can estimate the exact numbers of each cell type within each capture area. However, we are not able to identify which specific nuclei belong to each cell type. Thus, given that there are $n_{s,t}$ cells of type t at location s, we randomly assign $n_{s,t}$ nuclei as belonging to cell type t within the capture area.

For nuclei outside the capture areas, we have not collected any data. Thus, assume that the proportion of each cell type changes smoothly over the entire tissue. This assumption allows us to employe a Gaussian Process to impute the cell type proportions. Specifically, we let $f_t(\boldsymbol{\pi})$ denote the proportion of cell type t at location $\boldsymbol{\pi} = [\pi_x, \pi_y]$. We then assume the function $f_t(\boldsymbol{\pi})$ follows a Gaussian Process with the kernel function k. In other words,

$$\begin{bmatrix} f(\boldsymbol{\pi}_1) \\ \vdots \\ f(\boldsymbol{\pi}_n) \end{bmatrix} \sim MVN(\mathbf{0}, \boldsymbol{K} + \sigma^2 \boldsymbol{I}), \tag{20}$$

where $\boldsymbol{I}$ is the identical matrix, and the ij-th entry of matrix $\boldsymbol{K}$ is $k_{ij} = k(\boldsymbol{\pi}_i, \boldsymbol{\pi}_j)$. In this study, we use the squared exponential kernel, with the kernel function being expressed as

$$k(\boldsymbol{\pi}_i, \boldsymbol{\pi}_j) = \exp\left(-\frac{\left\|\boldsymbol{\pi}_i - \boldsymbol{\pi}_j\right\|_2^2}{2\theta^2}\right), \qquad (21)$$

where $\left\|\boldsymbol{\pi}_i - \boldsymbol{\pi}_j\right\|_2$ represents the Euclidean distance between the two locations, θ is the length-scale of the process. The parameters σ and θ are estimated by maximizing the likelihood. Therefore, we can use the Gaussian Process to estimate the proportion of each cell type in the neighborhood of each nucleus outside the capture areas. The estimated proportions at each location are normalized to ensure their sum equals 1. In this way, we assign a cell type to each nucleus outside the capture area by randomly sampling according to the estimated cell type proportions.

Through annotating the nuclei and inferring the cell boundaries, we obtain a pseudo single-cell resolution image that closely resembles the output of image-based spatial transcriptomics approach (Supplementary Fig. 19).

2 Generation of simulated spatial transcriptomics datasets

To create synthetic spatial transcriptomics datasets that closely resemble the actual dataset, we use the estimated number of each cell type at every location, as determined by Spotiphy, as the ground truth. Specifically, for generating the spatial expression at a given location s, we randomly sample $n_{s,t}$ cells of type t cells from the scRNA-seq data, for $t = 1, 2, \cdots, T$. We then merge all the scRNA-seq expressions. To enhance the resemblance of the synthetic expression to real data, we introduced three types of disturbance to the aggregated scRNA-seq data at each location: batch effect, artificial zero reads, and random noise. Specifically, let $\tilde{x}_{sg}$ denote the total count of gene

g at location s after aggregating the single cell gene expressions. We assume the batch effect parameter r_g independently follows the lognormal distribution with mean of 0 and sigma σ_{batch}. To introduce the artificial zero reads, we randomly sample δ_{sg} from Bernoulli distribution with mean of μ_δ. When δ_{sg} is 0, we set $\tilde{x}_{sg}$ to 0. Finally, random noise is introduced by sampling ε_{sg} independently from lognormal distribution with mean of 0 and sigma σ_{noise}. With these preparations, the generated expression of gene g at location s can be expressed as

$$\tilde{x}_{sg} = \tilde{x}_{sg} r_g \delta_{sg} \varepsilon_{sg}. \tag{22}$$

Since we aim to investigate the robustness of various deconvolution algorithm to disturbance, we consider three different levels of disturbance in this study. The parameters are chosen as follows. Small disturbance: $\sigma_{\text{batch}} = 0.1$, $\mu_\delta = 0.7$, $\sigma_{\text{noise}} = 0.05$. Medium disturbance: $\sigma_{\text{batch}} = 0.5$, $\mu_\delta = 0.4$, $\sigma_{\text{noise}} = 0.2$. Large disturbance: $\sigma_{\text{batch}} = 0.6$, $\mu_\delta = 0.3$, $\sigma_{\text{noise}} = 0.4$.

Supplementary Tables

Supplementary Table S1: Data processing details for all samples

Supplementary Table S2: Marker gene list of mouse brain scRNA reference

Supplementary Table S3: Ground truth and estimations of cell type proportions in AD sample

Supplementary Table S4: Ground truth and estimations of cell type proportions in the simulated sample

Supplementary Table S5: Astrocyte subtype differential expression analysis results

Supplementary Table S6: Astrocyte subtype GSEA analysis results

Supplementary Table S7: Microglia subtype differential expression analysis results

Supplementary Table S8: Microglia subtype GSEA analysis results

Supplementary Table S9: β-amyloid signal intensity for each spot from AD sample

Supplementary Table S10: DAM subtype differential expression analysis results

Supplementary Table S11: DAM subtype GSEA analysis results

Supplementary Table S12: Marker gene list of human breast scRNA reference

Supplementary Table S13: LumSec subtype differential expression analysis results

Supplementary Table S14: LumHR subtype differential expression analysis results

Supplementary Table S15: LumSec subtype GSEA analysis results

Supplementary Table S16: LumHR subtype GSEA analysis results

Supplementary Table S17: Ground truth and estimations of cell type proportions

Supplementary Table S18: Xenium and CosMx panels

Supplementary Table S19: Selected DEGs used in signature score definition
