# Supplementary material for: Spotiphy enables single-cell spatial whole transcriptomics across an entire section
Source: Nat Methods. 2025 Mar 12;22(4):724–36. doi: 10.1038/s41592-025-02622-5 (PMC11978521; doi:10.1038/s41592-025-02622-5)
Supplement: Supplementary file 2 — Reporting Summary [file 41592_2025_2622_MOESM2_ESM.pdf]

nature portfolio

Corresponding author(s): Jiyang Yu

Last updated by author(s): Dec 28, 2024

Reporting Summary

Nature Portfolio wishes to improve the reproducibility of the work that we publish. This form provides structure for consistency and transparency in reporting. For further information on Nature Portfolio policies, see our Editorial Policies and the Editorial Policy Checklist.

Statistics

For all statistical analyses, confirm that the following items are present in the figure legend, table legend, main text, or Methods section.

n/a	Confirmed	
☐	☒	The exact sample size (*n*) for each experimental group/condition, given as a discrete number and unit of measurement
☐	☒	A statement on whether measurements were taken from distinct samples or whether the same sample was measured repeatedly
☐	☒	The statistical test(s) used AND whether they are one- or two-sided<br>*Only common tests should be described solely by name; describe more complex techniques in the Methods section.*
☒	☐	A description of all covariates tested
☐	☒	A description of any assumptions or corrections, such as tests of normality and adjustment for multiple comparisons
☐	☒	A full description of the statistical parameters including central tendency (e.g. means) or other basic estimates (e.g. regression coefficient) AND variation (e.g. standard deviation) or associated estimates of uncertainty (e.g. confidence intervals)
☐	☒	For null hypothesis testing, the test statistic (e.g. *F*, *t*, *r*) with confidence intervals, effect sizes, degrees of freedom and *P* value noted<br>*Give P values as exact values whenever suitable.*
☐	☒	For Bayesian analysis, information on the choice of priors and Markov chain Monte Carlo settings
☒	☐	For hierarchical and complex designs, identification of the appropriate level for tests and full reporting of outcomes
☐	☒	Estimates of effect sizes (e.g. Cohen's *d*, Pearson's *r*), indicating how they were calculated

Our web collection on statistics for biologists contains articles on many of the points above.

Software and code

Policy information about availability of computer code

Data collection	Single-cell Sequencing alignments were done by Cell Rannger-arc v2.0.0.<br>Visium Sequencing alignments were done by Space Ranger v1.3.0.<br>CosMx data were collected through Services.<br>Xenium data were collected through Services, and analyzed by Xenum Explorer v1.3.0.

Data analysis	The Spotiphy package is available at https://github.com/jyyulab/Spotiphy. Usage of the code is introduced at https://jyyulab.github.io/Spotiphy/. Jupyter notebooks covering the analysis in this paper are available upon request.<br>Specific package versions used in Python:<br>Python 3.9.18; numpy 1.22.4; scipy 1.9.1; scikit-learn 1.2.2; Scanpy 1.9.3; anndata 0.10.3; opencv-python 4.8.1.78; torch 2.1.1; pyro-ppl 1.8.4; tensorflow 2.12.0; stardist 0.8.3.<br><br>Specific package versions used in R 4.1.3:<br>Seurat 4.3.0; Harmony 0.1.1; CellChat 1.5.0; InferCNV 1.3.3; NetBID 2.0; scMINNER 1.0.0. Dependencies have not been listed for brevity.<br><br>Package versions of benchmark methods:<br>Cell2location 0.1.3; Tangram 1.0.4; CARD 1.0; RCTD 2.2.1; cytoSPACE 1.0.6; iStar (no version number); Stereoscope 0.2.0; SPOTlight 1.10.0; MuSic, 1.0.0; SpatialScope 0.0.1; SpatialDWLS 4.0.0; Redeconve 1.1.1; CIBERSORTx https://cibersortx.stanford.edu/.

For manuscripts utilizing custom algorithms or software that are central to the research but not yet described in published literature, software must be made available to editors and reviewers. We strongly encourage code deposition in a community repository (e.g. GitHub). See the Nature Portfolio guidelines for submitting code & software for further information.

Data

Policy information about availability of data

All manuscripts must include a data availability statement. This statement should provide the following information, where applicable:

- Accession codes, unique identifiers, or web links for publicly available datasets
- A description of any restrictions on data availability
- For clinical datasets or third party data, please ensure that the statement adheres to our policy

The scRNA-seq datasets for mouse brains contain two parts: the mouse whole cortex and hippocampus scRNA-seq dataset is available at Allen Brain Map Atlas (portal.brain-map.org/atlases-and-data/rnaseq/mouse-whole-cortex-and-hippocampus-10x). The immune cell-enriched scRNA-seq dataset generated in this study is available at the Zenodo data repository under record number 10520022. The final scRNA-seq reference used in this study can also be found at the Zenodo data repository under record number 10520022. Additionally, the matched spatial transcriptomics (ST) datasets for WT and AD mouse brains, including Visium, Xenium, and CosMx, are available at the Zenodo data repository under record number 10520022. iscRNA datasets and simulated ST datasets used for evaluations are available at https://spotiphy.stjude.org.

The snRNA-seq dataset for astrocyte was downloaded from the ArrayExpress under accession number E-MTAB-11115. The scRNA-seq dataset for human breast cancer is downloaded from the Gene Expression Omnibus under accession number GSE195665. The three Visium datasets for human breast cancer are downloaded from the Zenodo data repository under record number 4739739. The scRNA-seq dataset for human lung is downloaded from Human lung cell Atlas (https://fetal-lung.cellgeni.sanger.ac.uk/scRNA.html). The Visium dataset for human lung cancer is downloaded from 10x Genomics (https://www.10xgenomics.com/datasets/human-lung-cancer-ffpe-2-standard). The Xenium dataset for human lung cancer is downloaded from 10x Genomics (https://www.10xgenomics.com/datasets/ffpe-human-lung-cancer-data-with-human-immuno-oncology-profiling-panel-and-custom-add-on-1-standard). The scRNA-seq dataset for human colorectal cancer is downloaded from Synapse under record number 26844071. The Visium dataset for human colorectal cancer is downloaded from 10x Genomics (https://www.10xgenomics.com/datasets/human-colorectal-cancer-11-mm-capture-area-ffpe-2-standard). The Xenium dataset for human colorectal cancer is downloaded from 10x Genomics (https://www.10xgenomics.com/datasets/ffpe-human-colorectal-cancer-data-with-human-immuno-oncology-profiling-panel-and-custom-add-on-1-standard). Eight additional datasets for deconvolution benchmarking are downloaded from github.com/QuKunLab/SpatialBenchmarking.

Research involving human participants, their data, or biological material

Policy information about studies with human participants or human data. See also policy information about sex, gender (identity/presentation), and sexual orientation and race, ethnicity and racism.

Reporting on sex and gender	N/A
Reporting on race, ethnicity, or other socially relevant groupings	N/A
Population characteristics	N/A
Recruitment	N/A
Ethics oversight	N/A

Note that full information on the approval of the study protocol must also be provided in the manuscript.

Field-specific reporting

Please select the one below that is the best fit for your research. If you are not sure, read the appropriate sections before making your selection.

☒ Life sciences ☐ Behavioural & social sciences ☐ Ecological, evolutionary & environmental sciences

For a reference copy of the document with all sections, see nature.com/documents/nr-reporting-summary-flat.pdf

Life sciences study design

All studies must disclose on these points even when the disclosure is negative.

Sample size	18 mouse samples, comprising 8 WT and 8 FAD models, were used for single-cell data profiling. One pair of mouse samples (WT_1 and FAD_1) was utilized across multiple spatial transcriptomics (ST) platforms. Sample size calculations were not performed. The sample size for scRNA-seq data was determined based on extensive prior experience with these technologies in mouse brains. Clustering analysis demonstrates its robustness to downsampling, indicating that the sample size is sufficient. Regarding ST data, each tissue section is capable of producing only one type of ST data. Because we are utilizing adjacent sections to generate matched datasets, we obtain only one data for every ST platform from each sample. Later clustering analysis also shows the sample size is sufficient.
Data exclusions	No data was excluded.
Replication	8 Replicates were gathered from both WT and FAD genotypes for single-nucleus data profiling. All samples were sequenced successfully and included in the final dataset. The final dataset had no batch effects.
Randomization	Randomization is not relevant to our study design as this is not a trial. There were no WT vs. FAD group comparison using scRNA-seq data.
Blinding	Prior to clustering, the researchers were blind to the mouse sample labels and genotype information. Unsupervised clustering was performed blind to the cell source or any other metadata that could reveal sample identity. Cell annotation was based on previously known marker genes.

Reporting for specific materials, systems and methods

We require information from authors about some types of materials, experimental systems and methods used in many studies. Here, indicate whether each material, system or method listed is relevant to your study. If you are not sure if a list item applies to your research, read the appropriate section before selecting a response.

Materials & experimental systems

n/a	Involved in the study	
☐	☒	Antibodies
☒	☐	Eukaryotic cell lines
☒	☐	Palaeontology and archaeology
☐	☒	Animals and other organisms
☒	☐	Clinical data
☒	☐	Dual use research of concern
☒	☐	Plants

Methods

n/a	Involved in the study	
☒	☐	ChIP-seq
☐	☒	Flow cytometry
☒	☐	MRI-based neuroimaging

Antibodies

Antibodies used	CD45 (ab10558, https://www.abcam.com/products/primary-antibodies/cd45-antibody-ab10558.html), CD11b (ab133357, https://www.abcam.com/products/primary-antibodies/cd11b-antibody-epr1344-ab133357.html)
Validation	The flow cytometry antibodies used in this study are widely used and have been validated by manufacturers.

Animals and other research organisms

Policy information about studies involving animals; ARRIVE guidelines recommended for reporting animal research, and Sex and Gender in Research

Laboratory animals	4-month-old 5XFAD, C57BL/6J, (all Jackson Laboratory, Bar Harbor, ME) mice were used in this study.
Wild animals	This study did not involve wild animals.
Reporting on sex	Sex was not considered in this study.
Field-collected samples	No field-collected samples were used.
Ethics oversight	All the experimental procedures in the animals were performed in accordance with the NIH Guide for the Care and Use of Laboratory Animals and all protocols, were approved by the St Jude Children's Research Hospital (protocol 542) IACUCS. Experiments were carried out in accordance with The Code of Ethics of the World Medical Association (Declaration of Helsinki) for animal experiments.

Note that full information on the approval of the study protocol must also be provided in the manuscript.

Plants

Seed stocks

Report on the source of all seed stocks or other plant material used. If applicable, state the seed stock centre and catalogue number. If plant specimens were collected from the field, describe the collection location, date and sampling procedures.

Novel plant genotypes

Describe the methods by which all novel plant genotypes were produced. This includes those generated by transgenic approaches, gene editing, chemical/radiation-based mutagenesis and hybridization. For transgenic lines, describe the transformation method, the number of independent lines analyzed and the generation upon which experiments were performed. For gene-edited lines, describe the editor used, the endogenous sequence targeted for editing, the targeting guide RNA sequence (if applicable) and how the editor was applied.

Authentication

Describe any authentication procedures for each seed stock used or novel genotype generated. Describe any experiments used to assess the effect of a mutation and, where applicable, how potential secondary effects (e.g. second site T-DNA insertions, mosiacism, off-target gene editing) were examined.

Flow Cytometry

Plots

Confirm that:

☐ The axis labels state the marker and fluorochrome used (e.g. CD4-FITC).

☐ The axis scales are clearly visible. Include numbers along axes only for bottom left plot of group (a 'group' is an analysis of identical markers).

☐ All plots are contour plots with outliers or pseudocolor plots.

☐ A numerical value for number of cells or percentage (with statistics) is provided.

Methodology

Sample preparation

Mice were deeply anesthetized with an Avertin and perfused with ice-cold 1xPBS (pH 7.4) to flush circulating blood cells from the brain. The exsanguinated brain was quickly removed, left hemisphere was collected by dissecting and placed in ice-cold HBSS (Gibco, Life Technologies NY, USA).The dissecting left hemisphere was used for profiling using 10x Genomics Chromium Single Cell Multiome ATAC + Gene Expression kit. A small piece of the tissue was flash frozen and used for direct nuclei isolation by following the 10X Genomics Demonstrated protocol Nuclei Isolation from Complex Tissues for Single Cell Multiome ATAC + Gene Expression Sequencing; CG000375 Rev A. Nuclei were counted by staining Trypan Blue on a hemocytometer and adjusted to a concentration of 1000 nuclei/uL. The remaining larger piece of the tissue sample was dissociated to yield single cells using papain-based dissociation solution on GentleMACS Octodissociator at 37 oC for 20 min. After filtering the dissociated sample, the neuronal and immune cells were separated from the unwanted myelin using density gradient centrifugation with 22% Percoll. The pellet containing microglia, lymphocytes, non-immune neuronal cells like astrocytes, oligodendrocytes, neuronal stem cells was washed in HBSS buffer and stained with CD45 (1:100) and CD11b (1:100) to enrich immune cell populations using FACS Aria III Cell Sorter. After isolating CD45+ & CD11b+ Microglia, CD45+ other Immune cells, and double negative neuronal cells were counted using AO/PI staining on Luna fl Cell Counter.

Instrument

BD FACSAria™ III Cell Sorter

Software

BD FACSDiva™ Software

Cell population abundance

15,000 Neuronal cells, 10,000 Microglia and all the cells of the immune population were mixed and used for nuclei isolation using 10X Genomics Demonstrated Protocol Nuclei Isolation for Single Cell Multiome ATAC + Gene Expression Sequencing; CG000365 Rev A. Nuclei were counted by staining Trypan Blue on a hemocytometer and adjusted to a concentration of 2000 nuclei/uL. Nuclei isolated from both pieces were mixed such that 5000 nuclei were targeted from nuclei isolated from enriched cell populations and 1000 nuclei were targeted from nuclei obtained directly from frozen section.

Gating strategy

To get the enriched immune cell populations using flow cytometry, start by creating a forward scatter (FSC) vs side scatter (SSC) plot to exclude debris and dead cells. Next, plot CD45 against SSC to identify and gate on CD45+ cells. From this CD45+ population, plot a CD11b vs SSC and gate on CD11b+ cells.

☐ Tick this box to confirm that a figure exemplifying the gating strategy is provided in the Supplementary Information.
